# Novel computational biology modeling system can accurately forecast response to neoadjuvant therapy in early breast cancer

**DOI:** 10.1186/s13058-023-01654-z

**Published:** 2023-05-10

**Authors:** Joseph R. Peterson, John A. Cole, John R. Pfeiffer, Gregory H. Norris, Yuhan Zhang, Dorys Lopez-Ramos, Tushar Pandey, Matthew Biancalana, Hope R. Esslinger, Anuja K. Antony, Vinita Takiar

**Affiliations:** 1 SimBioSys, Inc., 180 N La Salle St. Suite 3250, Chicago, IL 60601 USA; 2 Department of Radiation Oncology, University of Cincinnati, College of Medicine, Cincinnati, OH USA

**Keywords:** Breast cancer, Neoadjuvant therapy, Tumor volume prediction, Biophysical simulation, Virtual twin, Mathematical modeling

## Abstract

**Background:**

Generalizable population-based studies are unable to account for individual tumor heterogeneity that contributes to variability in a patient’s response to physician-chosen therapy. Although molecular characterization of tumors has advanced precision medicine, in early-stage and locally advanced breast cancer patients, predicting a patient’s response to neoadjuvant therapy (NAT) remains a gap in current clinical practice. Here, we perform a study in an independent cohort of early-stage and locally advanced breast cancer patients to forecast tumor response to NAT and assess the stability of a previously validated biophysical simulation platform.

**Methods:**

A single-blinded study was performed using a retrospective database from a single institution (9/2014–12/2020). Patients included: ≥ 18 years with breast cancer who completed NAT, with pre-treatment dynamic contrast enhanced magnetic resonance imaging. Demographics, chemotherapy, baseline (pre-treatment) MRI and pathologic data were input into the TumorScope Predict (TS) biophysical simulation platform to generate predictions. Primary outcomes included predictions of pathological complete response (pCR) versus residual disease (RD) and final volume for each tumor. For validation, post-NAT predicted pCR and tumor volumes were compared to actual pathological assessment and MRI-assessed volumes. Predicted pCR was pre-defined as residual tumor volume ≤ 0.01 cm^3^ (≥ 99.9% reduction).

**Results:**

The cohort consisted of eighty patients; 36 Caucasian and 40 African American. Most tumors were high-grade (54.4% grade 3) invasive ductal carcinomas (90.0%). Receptor subtypes included hormone receptor positive (HR+)/human epidermal growth factor receptor 2 positive (HER2+, 30%), HR+/HER2− (35%), HR−/HER2+ (12.5%) and triple negative breast cancer (TNBC, 22.5%). Simulated tumor volume was significantly correlated with post-treatment radiographic MRI calculated volumes (*r* = 0.53, *p* = 1.3 × 10^–7^, mean absolute error of 6.57%). TS prediction of pCR compared favorably to pathological assessment (pCR: TS *n* = 28; Path *n* = 27; RD: TS *n* = 52; Path *n* = 53), for an overall accuracy of 91.2% (95% CI: 82.8% – 96.4%; Clopper–Pearson interval). Five-year risk of recurrence demonstrated similar prognostic performance between TS predictions (Hazard ratio (HR): − 1.99; 95% CI [− 3.96, − 0.02]; *p* = 0.043) and clinically assessed pCR (HR: − 1.76; 95% CI [− 3.75, 0.23]; *p* = 0.054).

**Conclusion:**

We demonstrated TS ability to simulate and model tumor in vivo conditions in silico and forecast volume response to NAT across breast tumor subtypes.

**Supplementary Information:**

The online version contains supplementary material available at 10.1186/s13058-023-01654-z.

## Introduction

Despite pharmacologic and genomic progress in oncology, tumor heterogeneity hinders the ability to optimize therapy [1]. Molecular tumor characterization has advanced targeted therapeutics; despite this, expression of target biomarkers in any given patient does not necessarily ensure a predictable or durable response to therapy. Further compoudning challenges to optimizing therapy include the inability, a process of escalating or de-escalating therapy when it will result in better or comparable results with similar or fewer side effects, include the inability of large-scale trials with finite enrollment criteria to adequately address individual tumor characteristics (morphology [2, 3], vascularity [4, 5], location [6], tumor microenvironment [7]) and deliver personalized oncologic treatment recommendations that comprehensively address these variations in individual tumor conditions resulting from tumor heterogeneity.

Increasingly neoadjuvant therapy is being employed and International [8] and European [9] standard-of-care (SOC) guidelines for clinical practice continue to evolve. In the USA, these SOC regimens are designated by guidelines set forth by the National Comprehensive Cancer Network (NCCN) [10]. A rising number of SOC regimens, including new drug approvals and combinations of existing drugs, have become available for a single indication. These expanded coverage options for breast cancer are largely driven by an accelerated awareness of the complexity of tumor biology and a quest to increase offerings to the medical oncology community. What remains unknown is which SOC treatment will engender the best response in an individual patient and provide the best opportunity to achieve pathological complete response or a reduction in residual tumor burden. While some ineffectiveness may be attributed to suboptimal-guideline adherence [11, 12], even with adherence using clinical decision support systems to guide the decision-making process, clinical outcomes can vary within any sub-cohort given the same regimen. [13]

An innovative approach is needed to forecast treatment response and identify the likelihood of success of any chosen treatment. Accurately predicting tumor response affords numerous opportunities for the clinician in terms of increasing confidence around treatment selection, by providing information at the time of diagnosis to assist in understanding the treatment plan. This understanding will be particularly meaningful for patients who may be exposed to additional toxicity but would respond to an escalated regimen or those who may benefit from treatment de-escalation with comparable disease outcomes [14].

Currently, most methodologies appropriate clinical outcomes by stratifying patients according to risk of recurrence (ROR) and overall survival (OS) based on risk factors identified on retrospective analyses or -omics data [15–18]. However, modern day computational biology now affords a unique opportunity to use cutting-edge mathematical modeling to approximate in vivo biological processes in silico with a high degree of accuracy. Much of the field of perfusion kinetics [19, 20] has been derived from advances in medical imaging which now makes it possible to acquire dynamic, high spatial resolution images that have advanced multi-scale tumor modeling potential. These multi-scale computational models are now able to simulate and closely mirror fundamental cancer biological processes and predict spatiotemporal changes [21]. The next logical step in resolving the gap is in its predictive capacity to capture the dynamic environment on the time axis to forecast spatiotemporal changes of a patient’s tumor as it responds to chemotherapeutic agents.

Here, we perform an independent assessment of a previously validated biophysical tumor modeling platform [22], TumorScope Predict (TS). TS constructs a three-dimensional (3D), dynamic model of an individual’s tumor. By integrating modeling of tumor morphology and metabolism, the platform simulates biological processes and interactions that take place within the tumor microenvironment including vascularity, nutrient availability, drug delivery, sensitivity and resistance [22]. The integration of these complex interactions enables multimodal forecasting of tumor response over time to a given NAT.

In the current study, we further validate the platform’s ability to accurately evaluate and predict pCR, a surrogate marker for long-term outcome [23], in an independent breast cancer cohort receiving NAT for early-stage or locally advanced breast cancer (hereafter, referred to collectively as “early stage”). Additionally, we have undertaken efforts to further our understanding of the predictive capacity of TS, spanning residual tumor morphology, and ROR for breast cancer subtypes.

## Materials and methods

### Study population

Patients included in the study were age 18 or older, diagnosed with breast cancer, from September 2014 through December 2020, treated at a single institution who completed NAT with a SOC chemotherapy regimen, and had pre-treatment T1-weighted dynamic contrast enhanced magnetic resonance imaging (DCE-MRI). Patients with all subtypes and histology of breast cancer were included as well as those with bilateral breast tumors. One bilateral breast cancer case was analyzed as two separate tumors in the model, each with a distinct volumetric and pCR prediction; characteristics for the two tumors were analyzed to determine underlying biology. Retrospective data encompassing imaging and baseline diagnostic information were separated temporally into two time points: pre-treatment and post-treatment. The pre-treatment data (timepoint 1) was used for all analysis and computation. The post-treatment data (timepoint 2) was used for validation. Outcomes were single-blinded and run prospectively using timepoint 1. As de-identified patient data was used for the study; an institutional review board (IRB) exemption was granted.

A total of 81 tumors from 80 patients were analyzed. Patients with metastatic disease, those receiving neoadjuvant endocrine therapy alone, or those on clinical trials with experimental therapies or regimens were excluded from the study. The American Joint Committee on Cancer (AJCC) 8th edition staging system was used for all clinical and pathological assessments reported herein [15].

### Tumor segmentation and model design

**Fig. 1:**
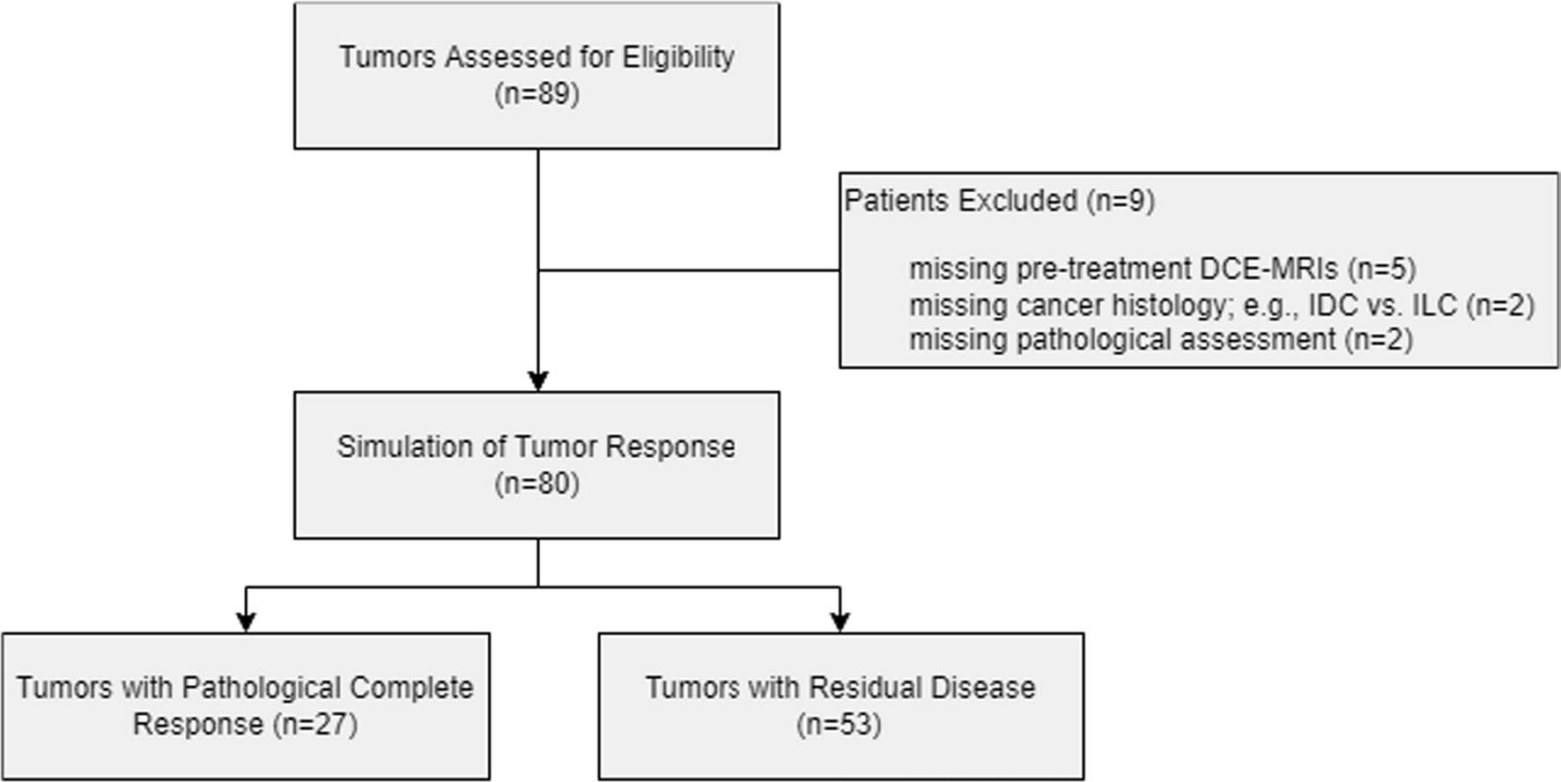
CONSORT flow diagram

Briefly, the previously described TS platform [22] combines artificial intelligence for tumor segmentation from the surrounding tissues with biophysical simulations to simulate the tumor response to therapy. It constructs a 3D virtual (in silico) tumor model of a patient (e.g., a virtual “twin”) that can be used to simulate how a patient will respond to a particular therapy using only baseline demographic, pathological, and medical imaging data. To create the 3D model, a pre-treatment DCE-MRI is processed using a semantic segmentation convolutional neural network (CNN)—a type of deep learning model with multiple successive layers that analyzes a neighborhood of voxels (e.g., pixels) and distils properties of them in increasingly abstract ways to assign a single class to each voxels [25]—to create a 3D representation of the breast tissues. In this case, the CNN is a multi-class volumetric residual UNet (e.g., a ResVNet [26], that examines each voxel in the MRI and classifies it as comprising primarily of tumor, vasculature, fibroglandular tissue, adipose, skin, or chest, producing a grid of cubic voxels that are 0.5 mm^3^ (the “spatial model”; see Fig. 1C, D) which forms the basis of the virtual twin. Briefly, the ResVNet consists of three encoding tiers, a bottleneck, and three decoding tiers and takes as input a 48 × 48 × 96 cubic voxel region with three channels (pre-contrast, early and late post-contrast) of the DCE-MRI. It was trained using fivefold cross-validation on the training set with a cross-entropy loss function; then, each fold was combined via weighted geometric means to produce the final model weights. None of the patients in this study were used in training the CNN. A recent publication describes the model training and validation [22].

Once the 3D model is created, the virtual twin is further personalized by incorporating patient demographic (e.g., age, race/ethnicity), and pathological characteristics (e.g., T (tumor size) and N (lymph node spread) stage, estrogen receptor (ER) percent staining, progesterone receptor (PR) percent staining, HER2 status, grade, histology type). These are used to select a mathematical model representing the patient’s tumor biology. Tumor biology algorithms were developed to describe the tumor’s requirements for nutrients (e.g., how quickly they consume nutrients and produce byproduct chemicals), interaction with neighboring healthy tissues (e.g., competition for, and cross feeding of, nutrients with fatty and glandular tissues), intrinsic growth rate (e.g., how fast the tumor doubles in size when consuming differing amounts of available nutrients), and susceptibility to drugs (e.g., pharmacodynamics). The combination of the tumor biology algorithms and the 3D spatial model constitutes the whole patient-specific virtual twin. The details of this process are described previously [22].

A completed virtual twin can then be simulated using the previously described biophysical model [22] with a NAT drug regimen. The simulation progresses by dosing drugs at regular intervals as defined by the National Comprehensive Cancer Network (NCCN) guidelines [10]. Drug dosing is captured by pharmacokinetic equations, which introduce the drug into the tissues according to a model based on the local vascular perfusion in a time dependent manner [22]. Vascular perfusion at each voxel is computed via a Tofts-like model that is parameterized from the DCE-MRI [27]. The simulation progresses by computing the local nutrient environment (i.e., concentration of metabolites like glucose, oxygen, alanine, etc.) and drugs at a specific time during treatment (see Fig. 1G). The growth/death rate of the tumor is computed as a function of the local nutrient and drug concentrations via the tumor biology model. The tumor size and shape are updated in response to the growth/death rate, and time is advanced by an increment of 1 h. This process is repeated until the entire course of treatment has been simulated (e.g., 60–180 days depending on drug regimen) [28–31]. The result is a 4D (3D + time) simulated picture of how the tumor responds to treatment.

In this study, the drug regimen prescribed for a patient by the physician was simulated. In total, 81 tumors in 80 patients were simulated. The final pre-surgery simulation tumor size and shape (see Fig. 1F) are used to determine whether the patient has pCR or RD as described in the next section.

### Model volume prediction and validation

Simulation volume for the model was defined as the sum of all volume fractions across all voxels that have disease. This sum was then multiplied by the physical volume of the voxel (in mm^3^) to generate the predicted, post-treatment volume.

For the radiologist-assessed volume, the tumor region of interest (ROI) was depicted (segmented) in 3D. Then, the number of voxels included in the ROI was summed and multiplied by the physical volume of the voxel (in mm^3^). Statistical analysis comparing the two models was then carried out.

### Statistical analysis

Virtual twins (of each individual tumor) were simulated with the physician-prescribed regimen. All analyses were performed on individual tumors; for patients with bilateral cancer, separate predictions were made for the tumor in each breast. Baseline demographics between the pCR and RD subgroups were compared using a chi-squared test (Table 1). Primary outcomes were the pCR/residual prediction along with predicted final volume for each tumor, which were both assessed at the timepoint 2 (e.g., the total simulated time duration was *timepoint 2-timepoint 1*). pCR predictions were compared to pathologist assessments reported in post-surgery pathology notes. Predicted volumes were compared to volumes segmented from post-treatment (timepoint 2, pre-surgery) MRIs. Segmented volumes were assessed by a board-certified radiologist (10 + years) specializing in breast cancer.

**Table 1:** Patient baseline demographics

	Overall (*n* = 80)	Residual (*n* = 53)	pCR (*n* = 27)	*p* value (corrected)
Age, mean (SD)	53.4 (10.2)	54.2 (9.8)	51.8 (11.1)	0.2
Height, mean (SD)	64.7 (2.4)	64.7 (2.4)	64.6 (2.5)	0.42
Race, *n* (%)				
African American	36 (45.0)	22 (41.5)	14 (51.9)	0.04
Asian	2 (2.5)	1 (1.9)	1 (3.7)	
Caucasian	40 (50.0)	29 (54.7)	11 (40.7)	
Hispanic	1 (1.3)		1 (3.7)	
Missing	1 (1.3)			
Histology, *n* (%)				
Invasive Ductal Carcinoma	72 (90.0)	49 (92.5)	23 (85.2)	< 0.008
Invasive Lobular Carcinoma	7 (8.8)	4 (7.5)	3 (11.1)	
Metaplastic Carcinoma	1 (1.3)		1 (3.7)	
Receptor status *n* (%)				
HR+/HER2+	24 (30.0)	15 (28.3)	9 (33.3)	< 1 × 10^–11^
HR+/HER2−	28 (35.0)	24 (45.3)	4 (14.8)	
HR−/HER2+	10 (12.5)	6 (11.3)	4 (14.8)	
TNBC	18 (22.5)	8 (15.1)	10 (37.0)	
Grade*, *n* (%)				
1	4 (5.0)	4 (7.5)		0.02
2	27 (33.8)	19 (35.8)	8 (29.6)	
2;3	4 (5.0)	2 (3.8)	2 (7.4)	
3	42 (52.5)	25 (47.2)	17 (63.0)	
Missing	3 (3.8)	3 (5.7)		
Tumor stage, *n* (%)				
T1	3 (3.8)	2 (3.8)	1 (3.7)	< 1 × 10^–3^
T2	57 (71.3)	37 (69.8)	20 (74.1)	
T3	19 (23.8)	13 (24.5)	6 (22.2)	
T4	1 (1.3)	1 (1.9)		
Nodal stage, *n* (%)				
N0	24 (30.0)	16 (30.2)	8 (29.6)	0.85
N1	48 (60.0)	32 (60.4)	16 (59.3)	
N2	8 (10.0)	5 (9.4)	3 (11.1)	
Regimen, *n* (%)				
AC-CFU	1 (1.3)	1 (1.9)		< 1 × 10^–8^
AC-T	1 (1.3)		1 (3.7)	
ddAC-T	9 (11.3)	8 (15.1)	1 (3.7)	
ddAC-wT	22 (27.5)	11 (20.8)	11 (40.7)	
T	1 (1.3)	1 (1.9)		
TC	10 (12.5)	9 (17)	1 (3.7)	
TCH	2 (2.5)	2 (3.8)		
TCHP	32 (40.0)	19 (35.8)	13 (48.1)	
wT-ddAC	1 (1.3)	1 (1.9)		
wTCarbo-ddAC	1 (1.3)	1 (1.9)		

The primary outcome metrics of positive predictive value (PPV), negative predictive value (NPV), sensitivity, specificity, and accuracy for pCR in the overall population—as well as in individual molecular subtypes [HR+/HER2−, HR+/HER2+, HR−/HER2+, and triple negative breast cancer (TNBC)]—were computed along with 95% confidence intervals (CI) estimated using the Clopper-Pearson exact binomial interval [32]. The accuracy of both residual tumor volume and percentage reduction in tumor volume as predictors of pCR was measured with the area under the receiver operating characteristic curve (AUROC). A pre-defined cutoff was used for binary pCR prediction; namely, a residual predicted tumor volume less than or equal to 0.01 cm^3^ or a greater than 99.9% reduction in predicted final tumor volume compared to diagnosis was considered pCR. This cutoff was pre-defined from feasibility studies of over 600 patients [22, 29, 33–36]. Predicted pCR was compared to standard pathologist assessments of pCR, defined as ypT0N0 per the AJCC staging system 8th edition [24]. To explore the ability of TS-predicted pCR to discriminate event-free survival (EFS), a long-rank test between survival curves for predicted pCR and predicted residual disease (RD) patients was used.

TS predictions were also correlated with radiographic response, assessed with the Pearson’s correlation coefficient with significance tested using the Fisher transformation. Statistics were computed with the Python package statsmodels v0.12.2. All statistical testing was done with *α* = 0.05, and false discovery correction was performed separately for descriptive analysis of demographic data and analysis of outcome metrics (sensitivity, specificity, correlation of volume with predictions, and survival outcomes)—using the Benjamini–Hochberg method with a false discovery rate of 0.05.

## Results

A total of 89 breast cancer patients who received NAT and had corresponding pre-treatment DCE-MRIs were identified at a single institution. Nine patients were excluded due to missing pre-treatment DCE-MRIs (5 patients), missing cancer histology (2 patients), or missing pCR calls (2 patients) (Fig. 1).

Eighty patients were eligible for inclusion in the generated for analysis (Table 1, Fig. 1), of which 36 (45.0%) were self-identified as African American women and 40 (50.0%) were self-identified Caucasian women. The median patient age was 53 years. Most tumors were high grade (54.4% grade 3) invasive ductal carcinomas (90%). The most common receptor subtype was HR+/HER2− (35%); additional receptor subtypes in the cohort were HR+/HER2+ (30%), TNBC (22.5%), and HR−/HER2+ (12.5%). The majority of patients were T2 (71%) and had either no spread to nearby lymph nodes (N0, 30%) or spread to 1–3 axillary lymph nodes (N1, 60%). Within this cohort, TNBC patients experienced higher rates of pCR, while HR+/HER2− patients had higher rates of RD (*p* < 1 × 10^–11^). Most of the pCR responses were observed in high-grade tumors (Table 1), consistent with other studies [37, 38] showing that higher grade (e.g., highly proliferative) tumors respond better to chemotherapy. Achievement of pCR varied across the different drug regimens that the patients received (*p* < 1 × 10^–8^).

The demographic and cancer characteristics of the population in this study were statistically different (*χ*^2^ test) when compared to a previous [22] study. Patients differed in racial/ethnic compositions, with a lower proportion of self-reported Caucasian patients (*p* = 0.011), a lower proportion of ductal versus lobular cancers (*p* = 0.005), a higher proportion of HR+/HER2+ and lower proportion of TNBC patients (*p* < 0.001). Moreover, this population had a higher proportion of grade 1 and 2 tumors (*p* < 0.001), a lower proportion of T1 versus T2 tumors (*p* = 0.004), and a lower proportion of N0 versus N1 tumors (*p* < 0.001).

**Fig. 2:**
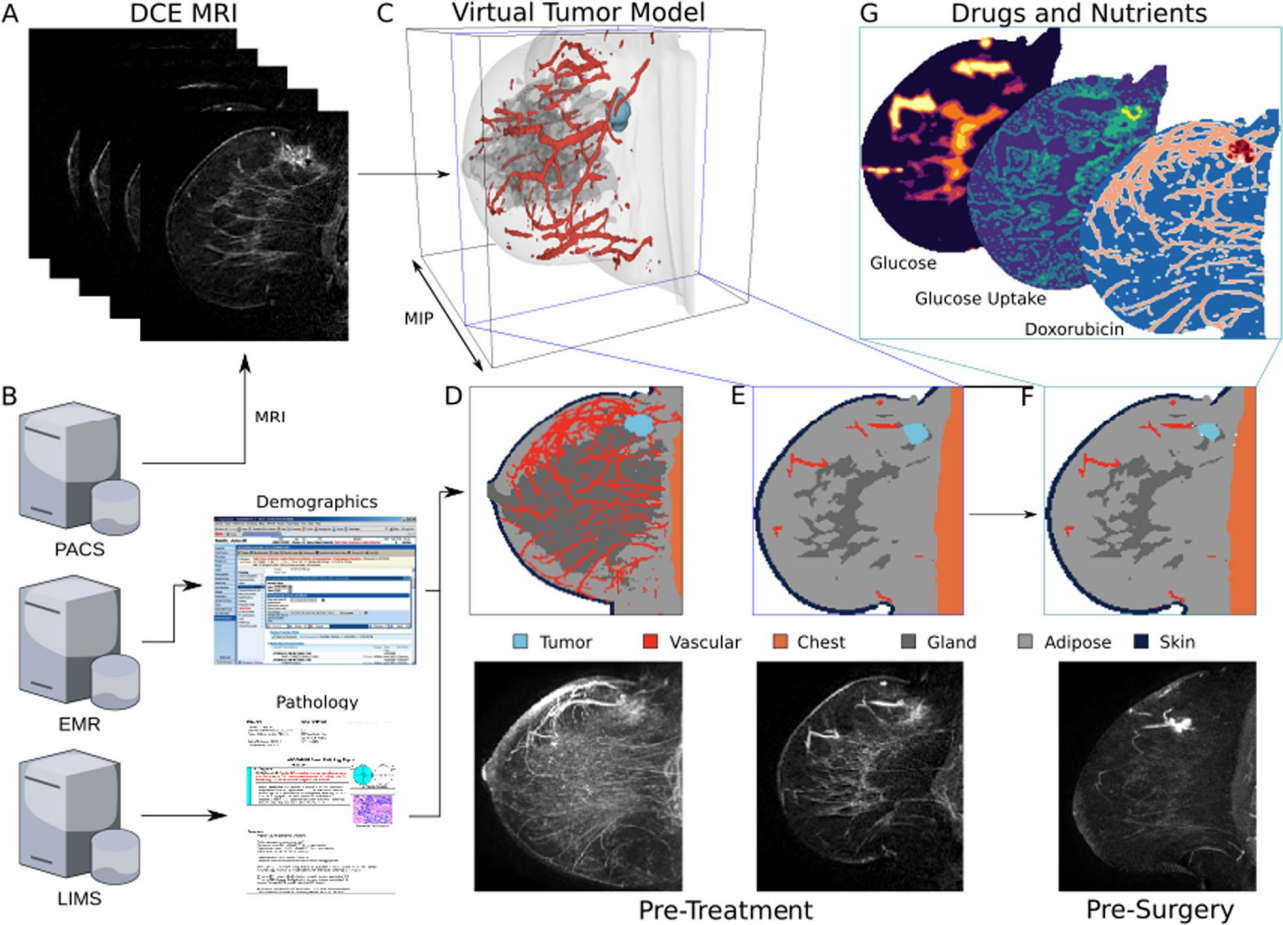
TumorScope Predict model. **A** DCE-MRI imaging data form the basis of a patient’s virtual tumor via a deep learning-based segmentation model that classifies each voxel as comprising primarily tumor, vascular, fibroglandular, adipose, skin, and chest “tissues”. **B** Along with imaging data extracted from the hospital’s PACS server, a patient’s demographic and pathology data are extracted from the EMR and LIMS servers, respectively, to create a unique profile of the tumor biology in the virtual tumor. **C** A rendering of a 3D virtual tumor model for a patient is shown, along with a comparison of the pre-treatment segmentation with the MRI as MIPs through the volumes (**D**) and as slices through the 3D volume at the point of greatest tumor area (**E**). The virtual tumor model is input into the TS simulation engine, which simulates the response to treatment longitudinally throughout treatment to the surgery date. This pre-surgery data can be compared directly to MRIs taken for surgery planning (**F**). **G** The TS platform simulates the spatial gradients of important drugs and nutrients, and how the tumor dynamically responds to them, capturing drivers of response

Previously acquired pre-treatment diagnostic data for these patients (demographic characteristics, drug regimen information, imaging (DCE-MRI), and pathology data) were input into TS (Fig. 2).

**Fig. 3:**
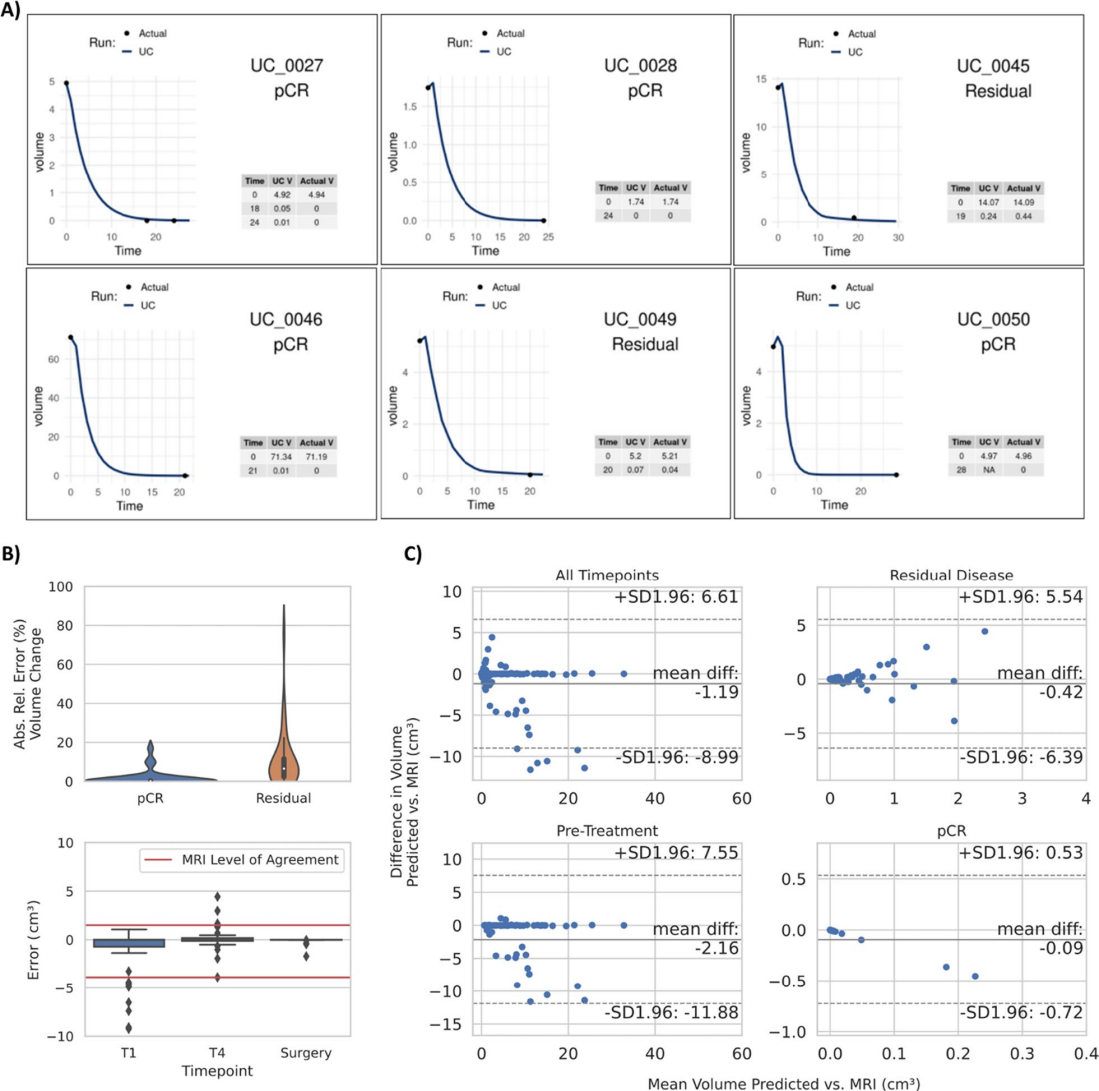
TumorScope Predict tumor volume predictions. **A** Representative figures of TS tumor volumetric response simulations. Each plot represents an individual patient where clinical (ground truth) volumetric measures (black dots) are compared to TS-simulated volume (blue continuous line). **B** Absolute relative error (%) in volume change between actual vs predicted pCR and residual volumes (top). Volume error (cm^3^) in the inter-treatment (T1 and T4) MRIs up to the time of surgery (bottom). Red lines indicate the MRI level of agreement [39]. **C** Volume comparisons between predicted and actual volumes are shown for all timepoints (top left), pre-treatment (bottom left), RD (top right) and pCR (bottom right)

TS then modeled the weekly volumetric response throughout the specific treatment regimen for each patient up to the point of surgery. Predictions of tumor volume strongly correlated with radiographic assessment of tumor volume for follow-up (*n* = 53) MRIs obtained after treatment for each patient (*r* = 0.53, *p* < 1.3 × 10^–7^; Fig. 3A, Additional file 1: Figs. S1 and S3). It should be noted that follow-up MRIs were used solely for validation, not as input to TS.

The mean absolute error in predicted tumor volume change for all follow-up MRIs was 6.57% demonstrating high accuracy of TS predictions (Table 2, Fig. 3B top and C). While error in the volume is difficult to interpret without a reference error rate, agreement between radiologically-assessed tumor size and pathologically-assessed tumor size has been examined [39]. Simulated volume errors were compared to the level of agreement (LOA) between breast MRIs and histopathology reported previously [39] (Fig. 3B bottom). For both inter-regimen and post-treatment MRI scans the mean volume error remained under 3.7% (Table 2) with most of the error distribution within the LOA.

**Table 2:** Absolute relative error in volume change and relative error in volume predictions of TumorScope Predict

	Absolute relative error (%)		Relative error (%)	
	Mean (std. dev.)	Median (MAD)	Mean (std. dev.)	Median (MAD)
All patients	6.57 (12.1)	1.45 (1.44)	1.38 (13.8)	− 0.02 (1.45)
Post-treatment (residual)	10.8 (14.8)	6.52 (4.62)	3.69 (18.0)	0.97 (5.55)
Post-treatment (pCR)	1.41 (3.90)	0.03 (0.03)	− 1.41 (3.90)	− 0.03 (0.03)

**Fig. 4:**
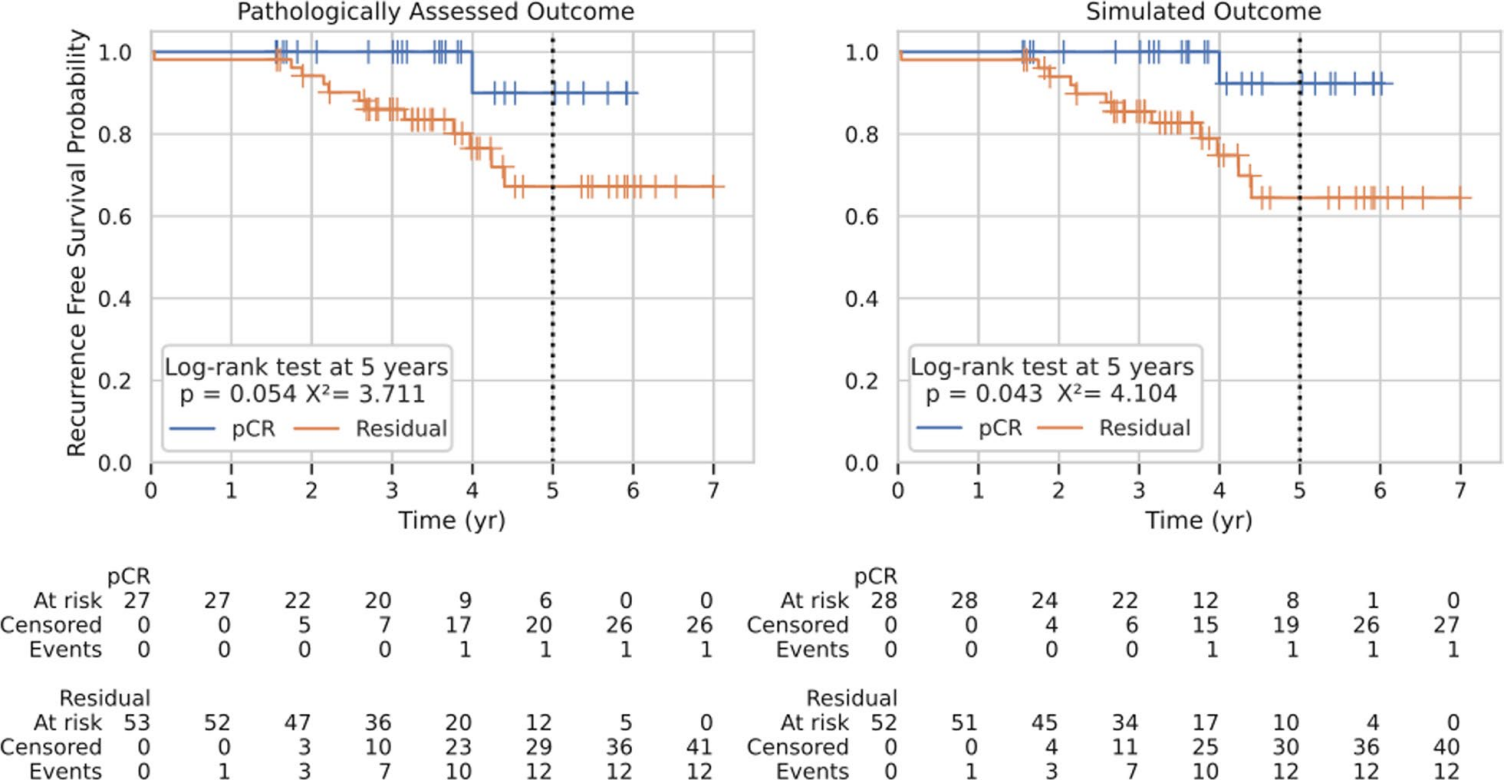
TumorScope Predict prognostic assessment. Graphs represent the probability of recurrence-free survival of patients that were clinically (left) evaluated for pCR (*n* = 27) or RD (*n* = 53) against TS predicted (right) pCR (*n* = 28) and RD (*n* = 52). TS predictions had similar prognostic value as clinical assessments. The zero-time point represents the date at which the patient had definitive surgery post-neoadjuvant treatment. Statistics were computed at 5-years post-surgery

Based on simulated tumor volumetric response, TS was then evaluated for its ability to predict pCR via the pre-defined tumor volume threshold 0.01 cm^3^ or percent reduction of 99.9% or greater. pCR was predicted in 28 patients, while RD was predicted in the remaining 52 patients (Fig. 4). The device predicted pCR/RD for 73 of 80 patients correctly, for an overall accuracy of 91.2 (95% CI 82.8–96.4%; Clopper-Pearson interval).

Device performance assessed via AUROC was found to be state-of-the-art (AUROC = 0.91; range 0.75–0.94). TS performance was robust throughout receptor subtypes, with the highest pCR prediction accuracy observed in TNBC patients at 93.8% (95% CI 55.5–99.8%) with a sensitivity of 90%, and the lowest seen in HR+/HER2− patients where the accuracy was 75% (95% CI 68–93.2%) corresponding to the lowest sensitivity of 50% (Table 3, Additional file 1: Fig. S2). The lower performance in HR+/HER2− patients was observed in a prior study [22], however the performance relative to other subtypes was not as extreme. The algorithm performance was also robust across chemotherapy regimens with a prediction accuracy of over 89% for both anthracycline (*n* = 33) and non-anthracycline (*n* = 47) regimens (Additional file 1: Table 1).

**Table 3:** TumorScope Predict outcome metrics, overall and subgroups

	Overall (*n* = 80)	TNBC (*n* = 18)	HR−/HER2+ (*n* = 10)	HR+/HER2− (*n* = 28)	HR+/HER2+ (*n* = 24)
AUROC (with volume threshold)	0.910	0.938	0.917	0.75	0.919
AUROC (with response threshold)	0.905	0.900	0.812	0.823	0.944
Accuracy	0.912 (0.828, 0.964)	0.944 (0.727, 0.999)	0.900 (0.555, 0.998)	0.893 (0.718, 0.977)	0.917 (0.730, 0.989)
Sensitivity	0.889 (0.708, 0.977)	0.900 (0.555, 0.998)	1.000 (0.398, 1.000)	0.500 (0.068, 0.932)	1.000 (0.664, 1.000)
Specificity	0.925 (0.818, 0.979)	1.000 (0.631, 1.000)	0.833 (0.359, 0.996)	0.958 (0.789, 0.999)	0.867 (0.595, 0.983)
PPV	0.857 (0.615, 0.958)	1.000 (NA, NA)	0.800 (0.309, 0.973)	0.667 (0.018, 0.996)	0.818 (0.460, 0.960)
NPV	0.942 (0.883, 0.972)	0.889 (0.500, 0.985)	1.000 (NA, NA)	0.920 (0.880, 0.947)	1.000 (NA, NA)

Accuracy represented as area under the receiver operating characteristic curve (AUROC) for the predicted pCR, computed either from a cutoff in the final volume or a cutoff in the response percentage (95% confidence intervals shown in parentheses; NA indicates confidence interval is undefined)

Next, probability of EFS was modeled to assess the prognostic ability of TS prediction of pCR versus RD compared to pathological assessment of pCR versus RD. TS-simulated pCR was associated with a 5-year ROR with a hazard ratio (HR) = − 1.99 [ − 3.96,  − 0.02] (95% CI; *p* = 0.043) while the clinically assessed pCR data had a HR = − 1.76 [ − 3.75,0.23] (95% CI; *p* = 0.054) demonstrating similar prognostic performance between TS predictions and pathological assessment (Fig. 4).

## Discussion

Advances in breast cancer oncology within the last decade have been largely in genomic profiling [15, 40, 41]. There is burgeoning recognition of the intricacies of tumor heterogeneity [42–44] that confounds expected treatment response to targeted pharmaceutical management, and newer oncologic insights are manifesting at an unprecedented rate [45, 46]. Existing unimodal technologies, including single cell biology [47, 48], organoids [49, 50] and spatial biology [47, 51] platforms, attempt to recapitulate varying aspects of multi-omics, biomarker expression patterns, and the complexity that stems from cells organizing and interacting in the 3D tumor microenvironment. Here, we perform a study of an independent cohort using a previously validated biophysical platform, TumorScope Predict. Predicated on mathematical modeling algorithms that incorporate dynamic, high-resolution imaging [52–56], this technology confers capacity to identify and manage tumor heterogeneity, such as region-specific vascular density, perfusion, simulated nutrient availability and simulated drug delivery to forecast tumor response on a case-by-case basis (see *Tumor Segmentation and Model Design*).

While traditional radiomics, the quantitative analysis of medical images, is a field of active investigation and interest, it relies on static 2D or 3D images for analysis. For example, a multivariate study [57] using MRI to predict pCR found that for all patients receiving NAT, pCR prediction failed to reach an AUROC over 0.660 [57], suggesting only modest gains in prediction performance. Radiomic features were only considered for prediction which may also explain lower performance. The addition of pre-treatment clinical information with machine learning increases the accuracy of pCR predictions [58], however, this approach remains suboptimal as it still fails to account for tumor progression and response throughout therapy. Even in vitro methods, such as organoids and in vivo methods, such as xenografts, which explore the behavior of living cells in response to a variety of targeted therapies, still lack the fundamental capacity to analyze tumor heterogeneity and complexity in relation to the dynamic communication and interplay between genetically or transcriptionally distinct regions of a tumor. Instead, both organoids and xenografts approach tumors as essentially a series of clonal experiments, without incorporating the higher-order behavior of a structurally diverse tumor. Thus, these methods do not truly address the heterogenous nature of the tumor that is thought to be a critical driver of therapy response.

The TumorScope technology works by amalgamating the fields of perfusion kinetics and computational biology to simulate the biophysical interactions between the tumor, surrounding tissues, nutrients and drugs in a 3D model. The inputs for the technology include non-invasive high-resolution image acquisition (DCE-MRIs) and standard of care pre-treatment information including demographics, pathological report data (including available molecular markers). Once basal conditions are established, the platform simulates physician-chosen drug regimens with fundamental in vivo cancer biological processes (e.g., metabolic activity) in a multi-scale biophysical computational model over the axis of time to create an adaptive, 4D model (3D model over time).

Precedence for this technology exists in preclinical models; Zangooei et al. demonstrated that microscale computed tomography (microCT) enabled computational simulations (multi-scale modeling) to recapitulate the dynamics of tumor growth in a rodent model [21]. In the current study, we found that TS-simulated tumors, as compared with radiographically-assessed tumors from MRIs, demonstrated highly accurate prediction of both tumor volume and percent change in tumor volume throughout therapy, with low median absolute error (Fig. 3, Table 2). Tumor volume has been used in several approaches to assess clinical outcomes either as an independent prognostic indicator [59] or in combination with other prognostic indicators such as pCR [60], residual cancer burden (RCB) [61], RFS [62, 63], progression-free survival (PFS) [64] and OS [64–67]. Previous studies have used initial volume parameters from 2D images obtained from medical images (MRIs and PET/CT scans) to predict long-term outcomes (pCR [68, 69] and RFS [62, 63]) in breast cancer [62, 63] lung [68, 69] and rectal cancer [70]. However, the accuracy of these methodologies has only reached an AUROC ranging from 0.62 [70] to 0.73 [69]. The TS platform uses multi-scale multimodal approach to predict the tumor’s response over time; attained pCR predictions with predictive accuracy ranging from 0.893 to 0.944, associated with an overall AUROC of at least 0.91. Importantly, overall performance of TS was robust in all breast cancer subtypes, indicating the tool may offer value in the NAT decision-making process. This substantial improvement relative to existing technologies not only advances the field of precision oncology, but additionally increases the likelihood of meaningful impact on clinical care.

More importantly, these volume predictions are forecasted based on physician-chosen drug dose and timing of standard of care chemotherapeutic regimens; newer agents (immunotherapy, antibody–drug conjugates) are actively being added into the platform’s drug repertoire to accommodate the changing landscape of pharmaceutical management of breast cancer. TS demonstrated high fidelity and accuracy near or above 90% in prediction of tumor response to both anthracycline and non-anthracycline based regimens. As anthracyclines have been a major target for de-escalation of therapy, given their known cardiotoxicity and association with secondary leukemia, these results are encouraging to inform decision-making and assist with understanding response to physician-chosen regimen. The proposed biophysical modeling approach thus confers domain over an expanse of clinical support areas ranging from de-escalation to prognostic predictions related to tumor volume such as pCR, RD and associated RFS.

While pCR at the clinical trial cohort level may not be generally indicative of an overall superior therapy [23, 71], it has proved to have meaningful prognostic value for individual patients, particularly for the TNBC and HER2+ subtypes [72]. TS-generated predictions of RFS derived from pCR mirrors RFS from clinically assessed pCR (Fig. 4). As TS predictions [23, 73, 74] rely on volume-based metrics (tumor volume rendered in 3D, changes in the tumor over time in 4D), these outputs provide insights on residual disease including morphology and volume. Thus, TS has the capacity to capture residual cancer burden (RCB) [73] in breast cancer, as well as potential for use in other cancers with the Response Evaluation Criteria in Solid Tumors (RECIST) [75] criteria. Given these outputs, volume-based tumor responses can be stratified into risk categories and assist in evaluating long-term prognosis.

There are certain limitations to our model. Foremost, prediction of pCR using TS is performed on analysis of the primary tumor only; no axillary lymph node disease is considered. However, studies have shown that lymph node histology is similar to that of the primary tumor; therefore, modeling the primary tumor could be representative of the actual tumor response [76]. Indeed, Fayanju et al. found that in a study of over 20,000 patients, only 1.5% of patients achieved pCR in the primary tumor but not the lymph nodes [77], and thus, one might anticipate that the error in overpredicting pCR would be small. Also, it depends on the availability and quality of the medical images. Not all patients are indicated to receive a pre-treatment DCE-MRI scan. Given that roughly 5.6% (5/89) of patients were excluded due to a missing pre-treatment MRI, this is hurdle for use, and potentially a source of bias in the study. Additionally, MRI quality will depend on the machines and techniques used and can affect the precision of the technology. Our platform models NAT per standard-of-care recommended regimens, and in this assessment potential treatment interruptions, delays and changes in medication may not have been considered. Finally, the nature of a single site study with a limited number of patients has resulted in wide confidence intervals; however, our results remain consistent with TS performance observed in concurrent validation studies [22, 33] with a multi-center analysis that addresses a much larger cohort of patients, currently underway. One assumption of the study is that multiple tumors in a single patient are independent, which may bias the statistics. One African American patient with HR+/HER2− invasive ductal carcinoma had bilateral cancer. These tumors differed only in that the left tumor was grade 2, while the right was grade 3. Both tumors were predicted (correctly) to have residual disease, which may slightly skew the statistics for the HR+/HER2− cohort. Despite any limitations imposed by these constraints, TS performance was robust across all subtypes. While the focus of this validation study is breast cancer, the potential to use biophysical simulation modeling approaches for other solid tumors is an area of active investigation for our team with encouraging preliminary results.

This validation study demonstrates that TS accurately predicts patient-specific tumor volume response to NAT across all breast cancer subtypes. These capabilities allow the platform to predict pCR in a patient-specific manner, which could in turn be used to optimize chemotherapy regimens and escalation/de-escalation decisions, predict downstaging, and inform the physician–patient discussion. By predicting tumor volume across the timescale of cancer therapy, TS offers predictive capacity with regards to pCR, RCB and EFS and is a useful technology for adjuvant therapy planning. Changes in volume, morphology, and particularly size (diameter) derived from the simulations in the current study with associated prognostic metrics can be used in patients diagnosed with other types of solid tumors, advancing precision oncology across a spectrum of disease.

While this and previous studies demonstrate method performance in a limited set of independent centers, a broader multi-center validation consisting of a significantly larger patient population is necessary to account for patient and clinical care variability seen in current practice.

## Supplementary Information

**Additional file 1.** Supplementary Tables and Figures.

## Data Availability

The data generated in this study are available within the article and its supplementary data files.

## Abbreviations

AUROC: Area under the receivers operating curve
DCE-MRI: Dynamic contrast enhanced magnetic resonance imaging
EFS: Event-free survival
ER: Estrogen receptor
HER2: Human epidermal growth factor receptor 2
HR+: Hormone receptor positive
HR−: Hormone receptor negative
HR: Hazard ratio
IRB: Institutional review board
NAT: Neoadjuvant therapy
OS: Overall survival
pCR: Pathological complete response
PFS: Progression-free survival
PR: Progesterone receptor
RCB: Residual cancer burden
RD: Residual disease
RECIST: Response evaluation criteria in solid tumors
ROR: Risk of recurrence
SOC: Standard-of-care
TS: TumorScope Predict
