# Supplementary material for: Novel computational biology modeling system can accurately forecast response to neoadjuvant therapy in early breast cancer
Source: Breast Cancer Res. 2023 May 10;25:54. doi: 10.1186/s13058-023-01654-z (PMC10170712; doi:10.1186/s13058-023-01654-z)

**Supplemental information**

**Title:** **Novel Computational Biology Modeling System Can Accurately Forecast Response to Neoadjuvant Therapy in Early Breast Cancer**

**Authors:** Joseph Peterson^1*^, John A. Cole^1*^, John Pfeiffer^1^, Greg Norris^1^, Yuhan Zhang^1^, Dorys Lopez-Ramos^1^, Tushar Pandey^1^, Matthew Biancalana^1^, Hope R. Esslinger^2^, Anuja K. Antony^1^, Vinita Takiar^2^

**Affiliations:**

^1^SimBioSys, Inc., Chicago, IL

^2^Department of Radiation Oncology, University of Cincinnati, College of Medicine

*Shared first co-author

**Running title:** Novel technology forecasts tumor response to neoadjuvant therapy

**Supplemental Figures**

**Supplemental Table 1. TumorScope pCR prediction performance for anthracycline-containing and non-containing regimens.**

|  | **n** | **Accuracy**  **(95% CI)** | **Sensitivity**  **(95% CI)** | **Specificity**  **(95% CI)** |
| --- | --- | --- | --- | --- |
| **Anthracycline containing** | 35 | 0.939  (0.798, 0.993) | 0.946  (0.546, 0.981) | 1.000  (0.832, 1.000) |
| **Non-anthracycline containing** | 45 | 0.894  (0.769, 0.965) | 0.929  (0.661, 0.998) | 0.879  (0.718, 0.966) |

**Supplemental Figure 1. Actual vs. predicted tumor volume comparisons.** Each plot corresponds to an individual patient. Black dots represent the ground truth radiographic volume (obtained from MRIs), while the blue lines represent the simulated volume throughout therapy.


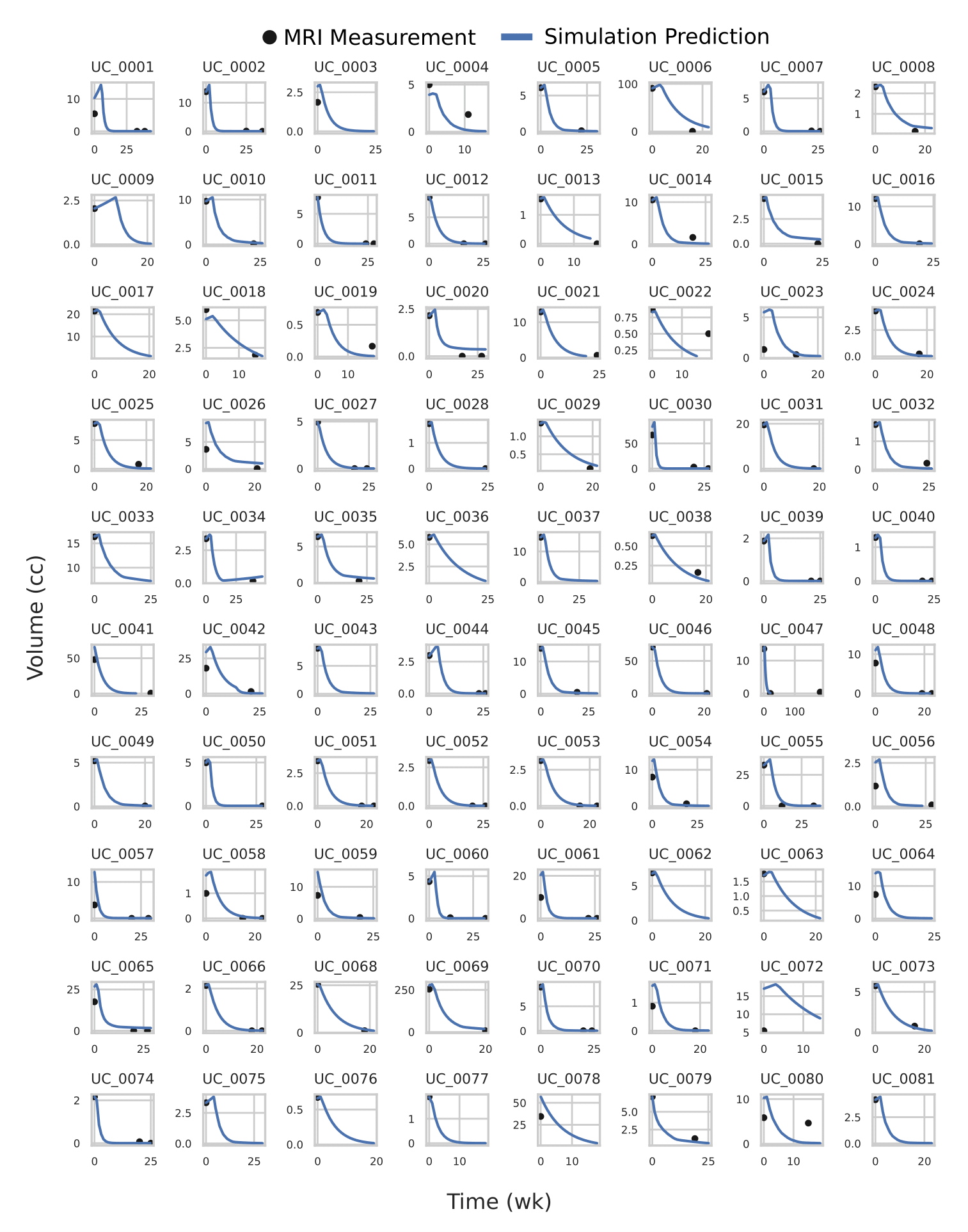


**Supplemental Figure 2. Receiver operating characteristic (ROC) curves showing area under the curve (AUROC) values for TumorScope predictions in different breast cancer subtypes.** TumorScope performance for volume and response percentage is represented as ROC curves for overall population (black) and stratified by tumor subtype: HR+/HER2-, TNBC, HR+/HER2+ and HR-/HER2+. TNBC had the highest volume performance (AUROC=0.938) and HR+/HER2+ the highest response performance. While HR+/HER2- had the lowest volume performance (AUROC=0.750) and HR-/HER2+ the lowest response performance (AUROC=0.812).


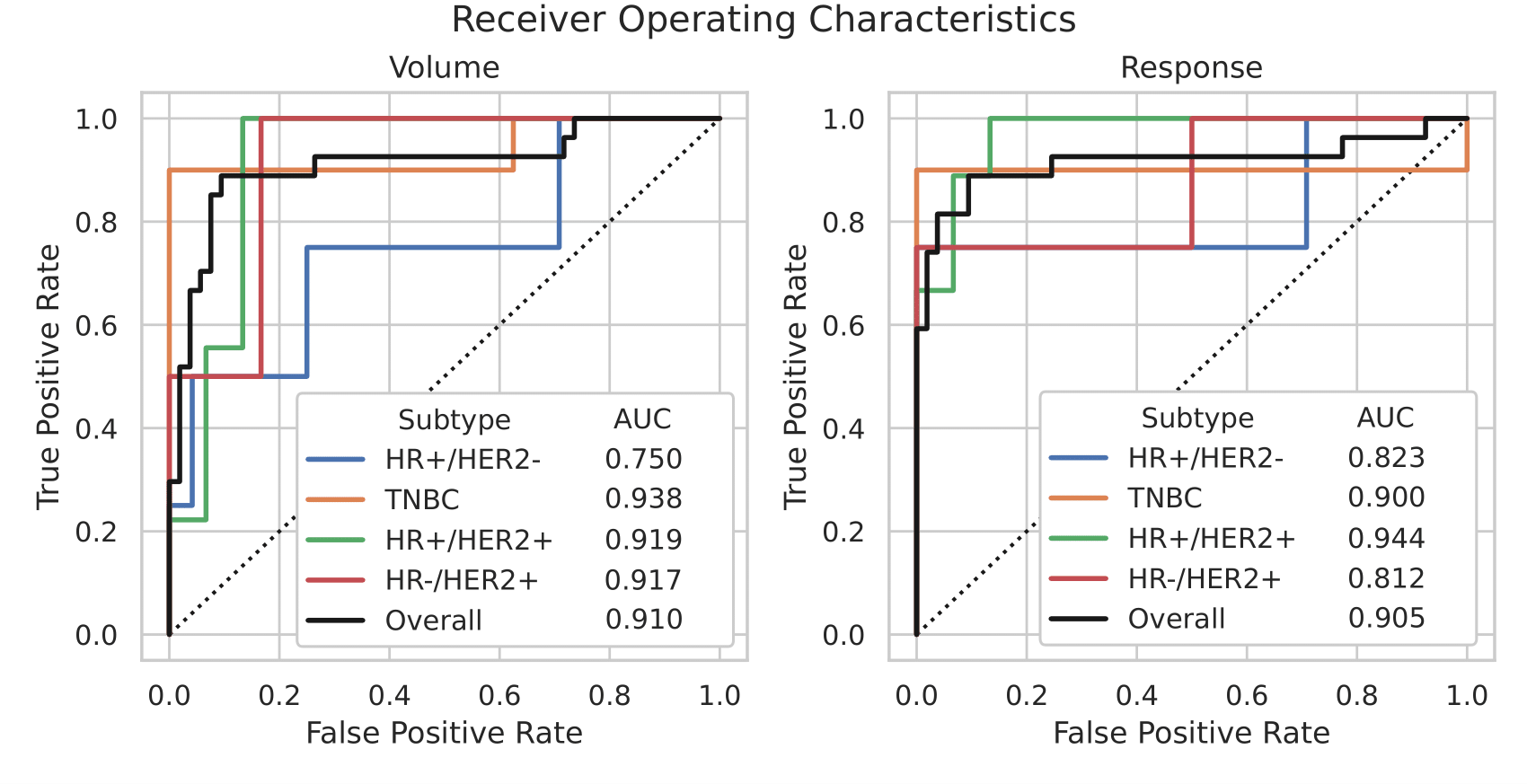


**Supplemental Figure 3. TumorScope predictions of tumor volume and morphology overlaid on pre- and post-treatment MRIs.** Panels show a representation of the pre-treatment tumor segmentation (top) and simulated post-treatment (bottom) tumor volume (red) overlayed on the corresponding MRI. Both the sagittal (left) and transverse (right) maximal intensity projections (MIPs) are shown for clarity. Patients that achieved pCR are shown in (A), while patients with RD are shown in (B).

**A) pCR**


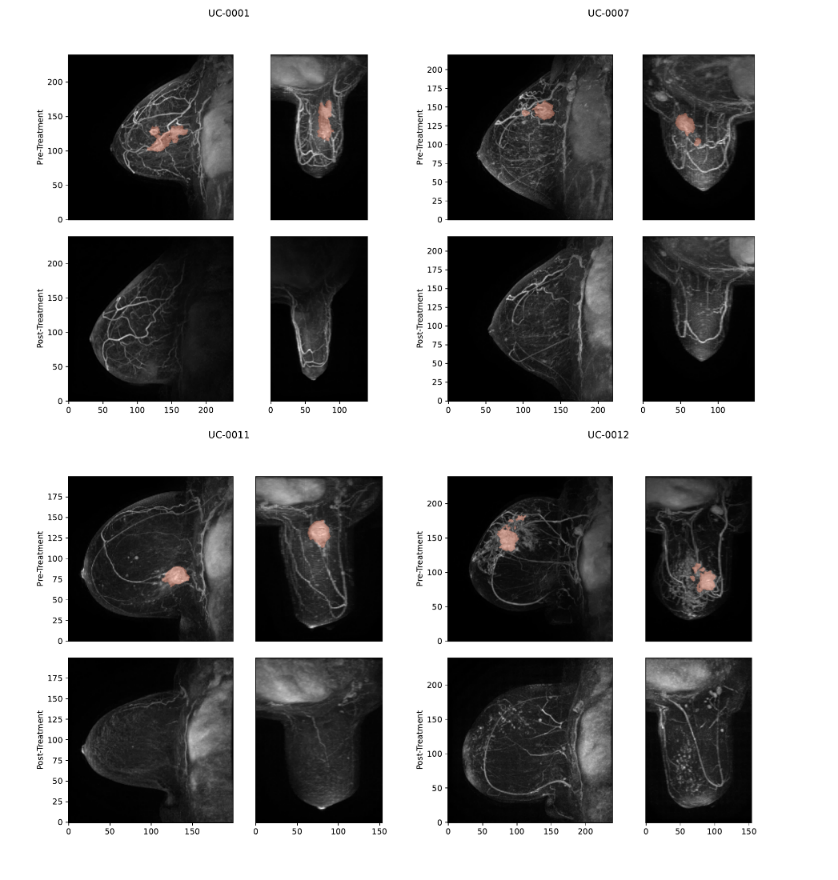


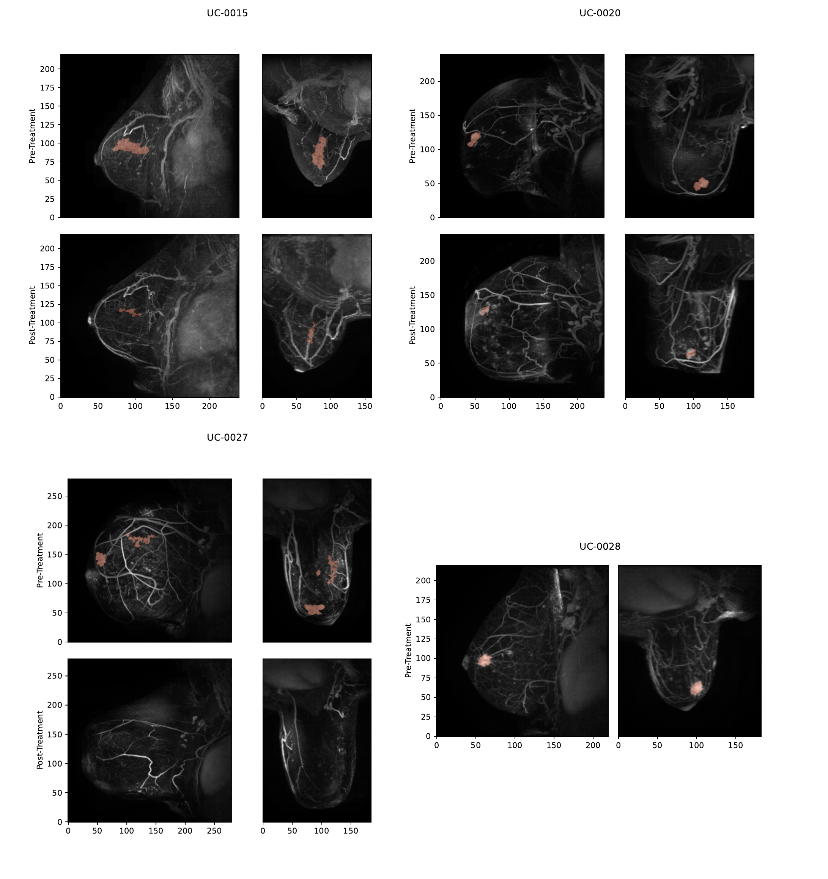


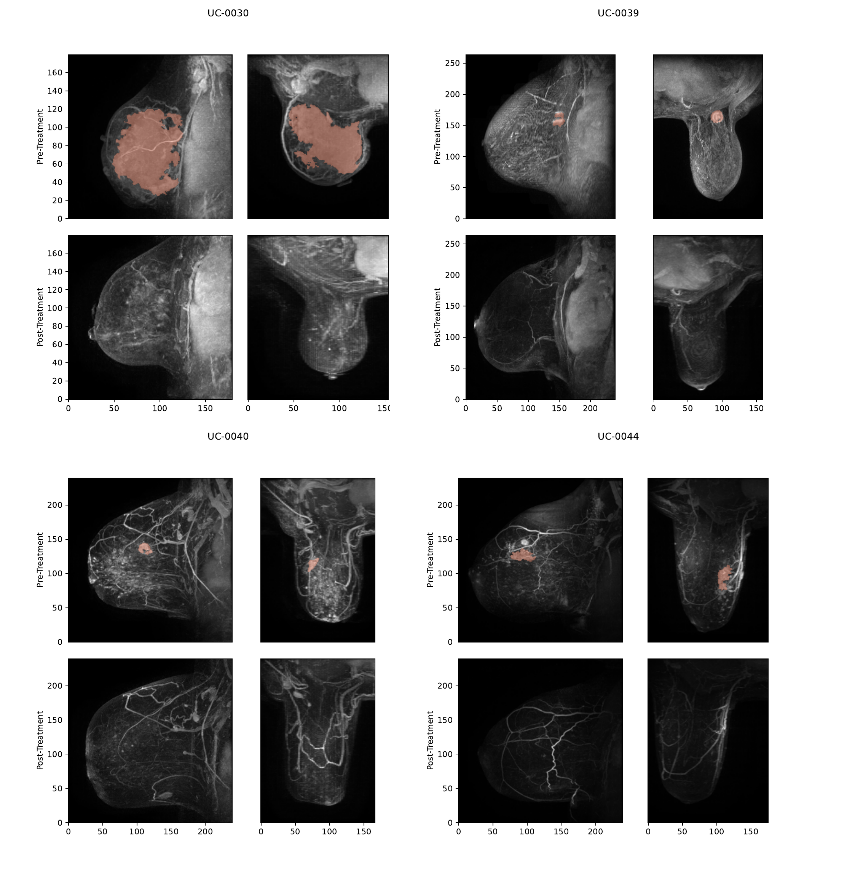


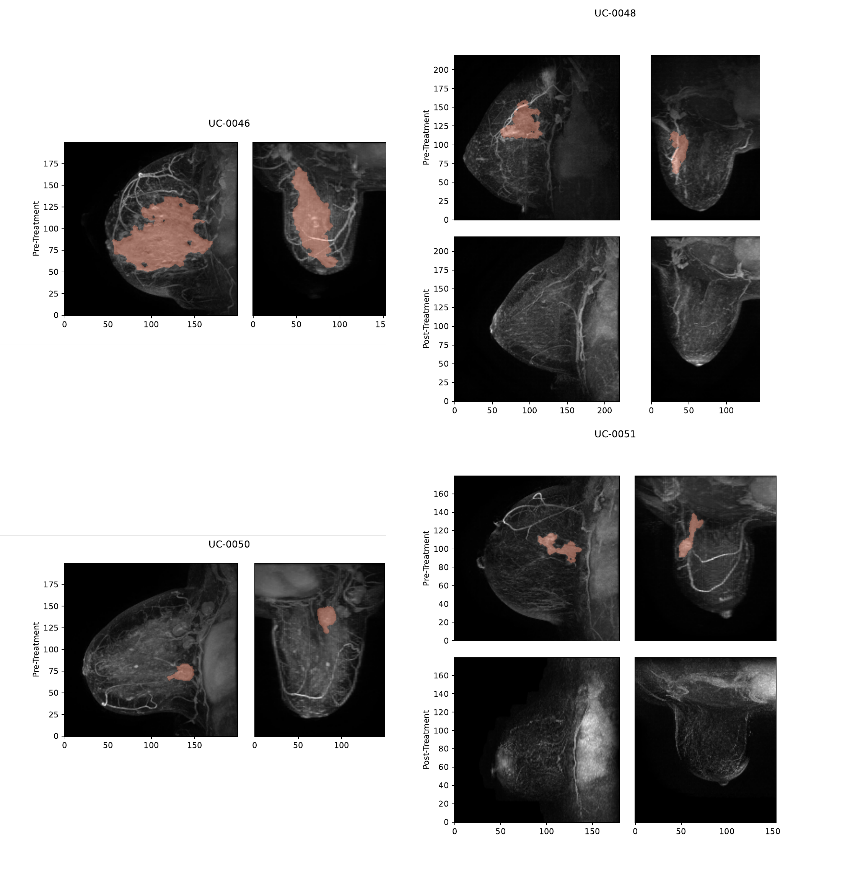


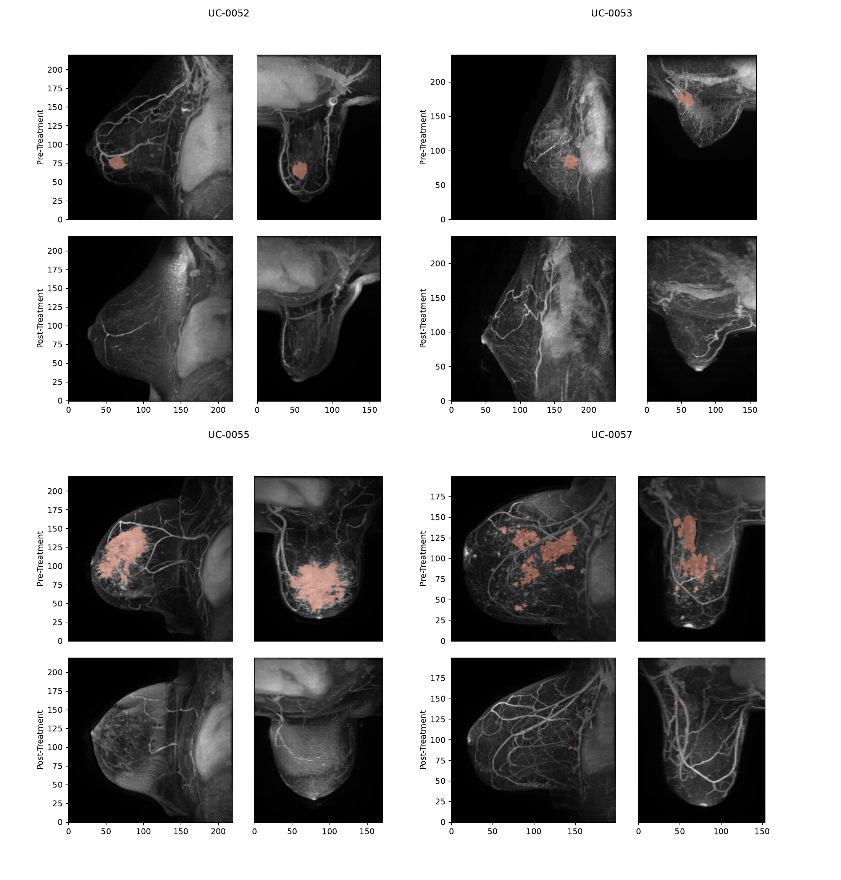


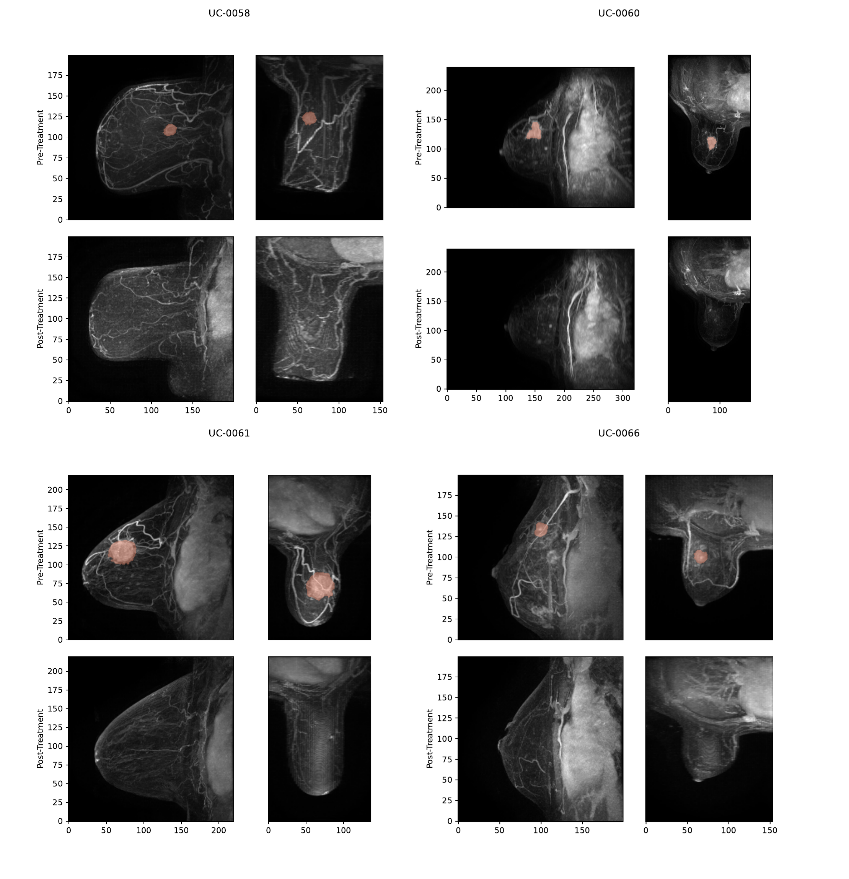


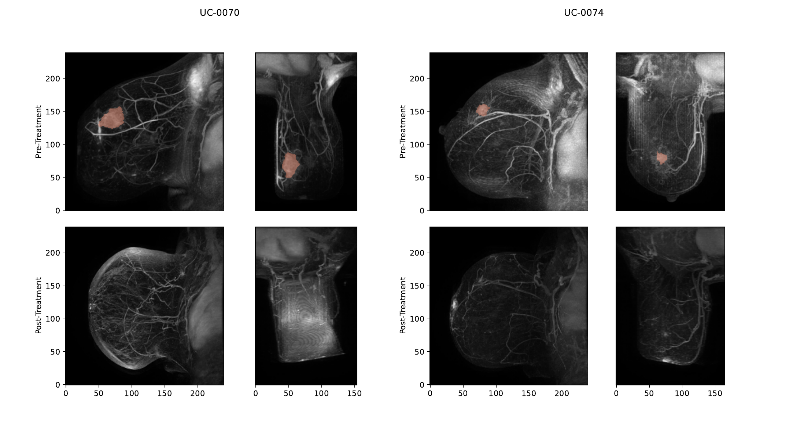


**B) Residual**


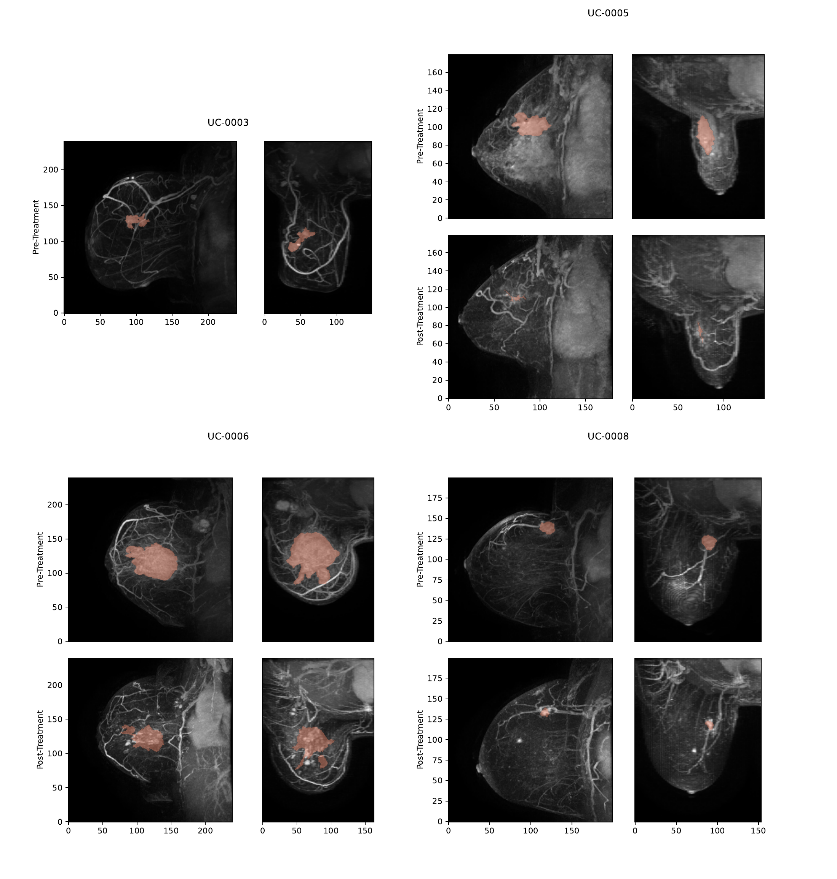


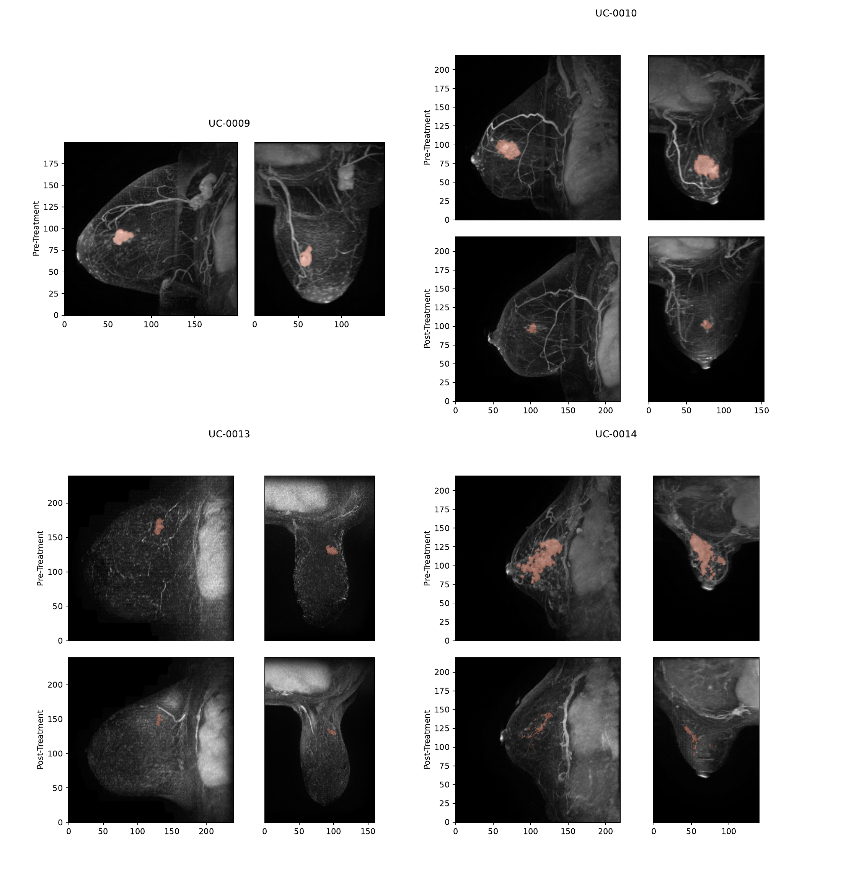


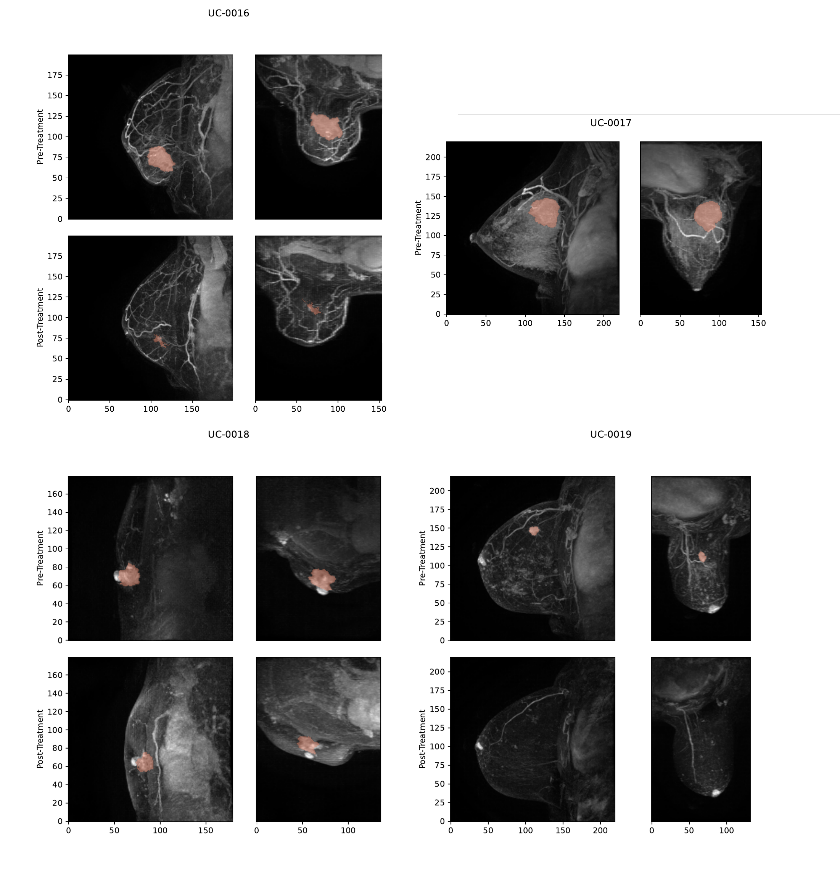


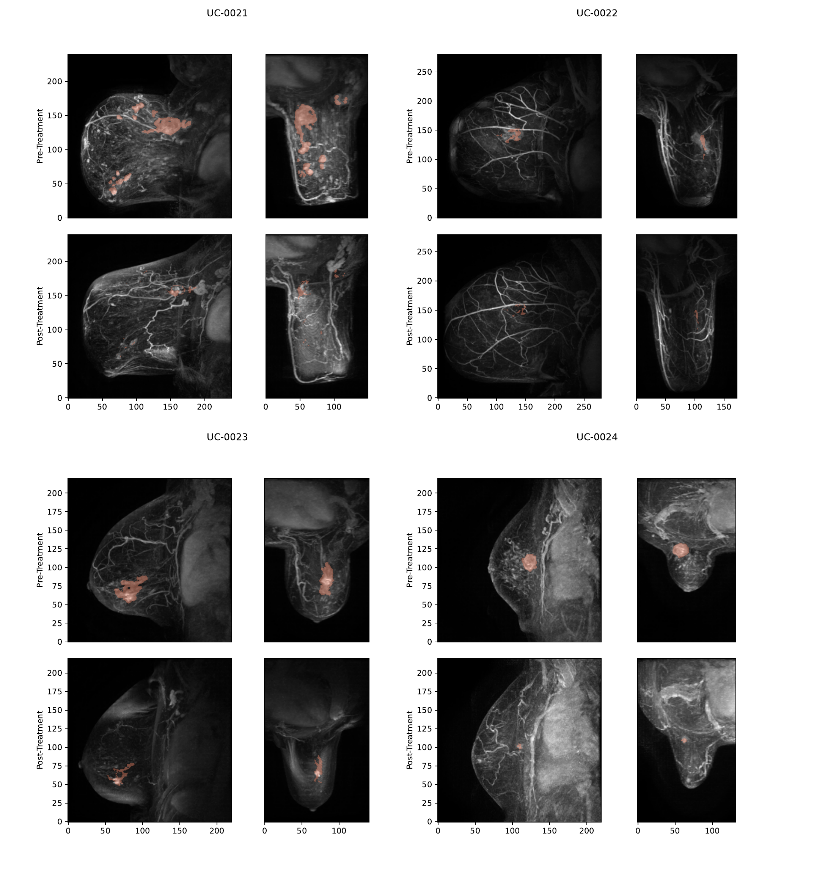


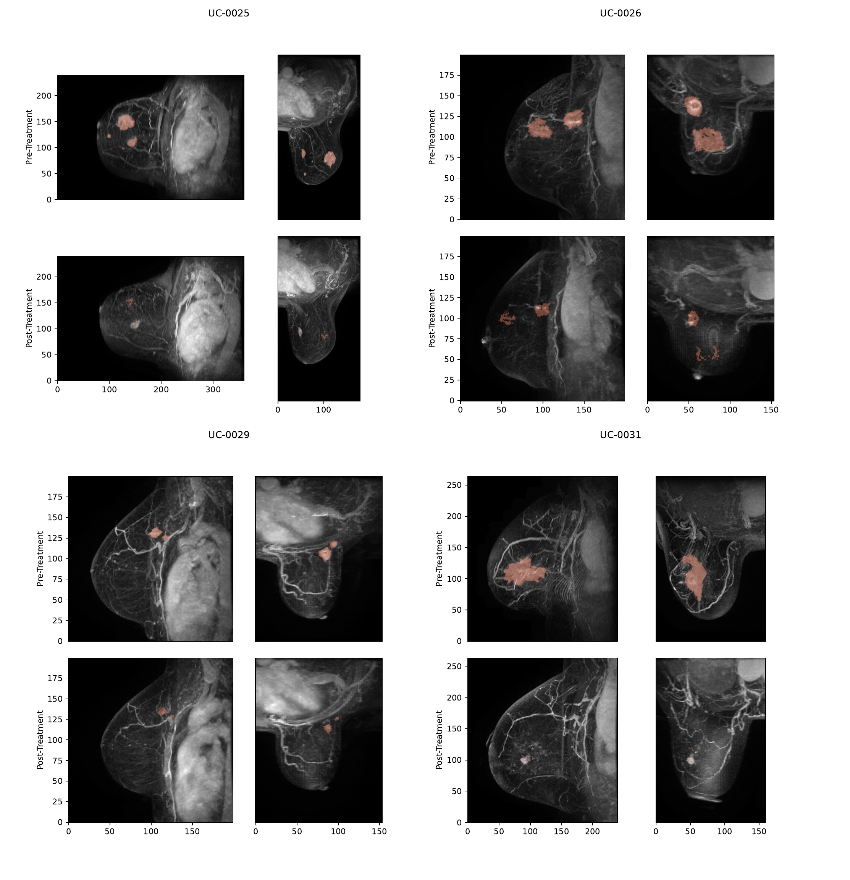


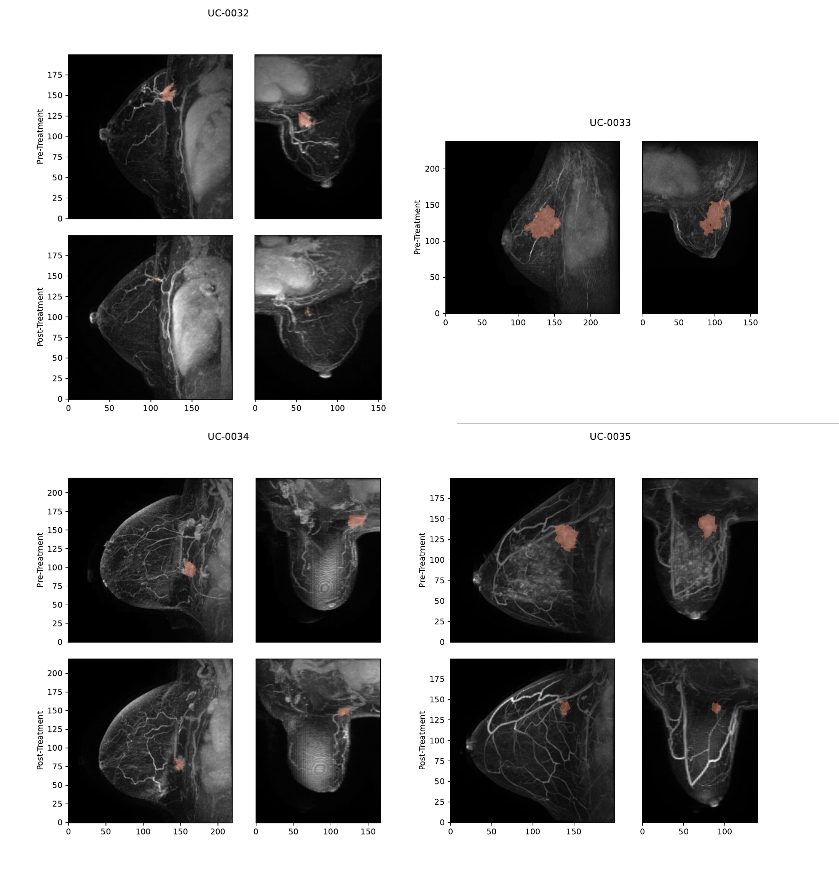


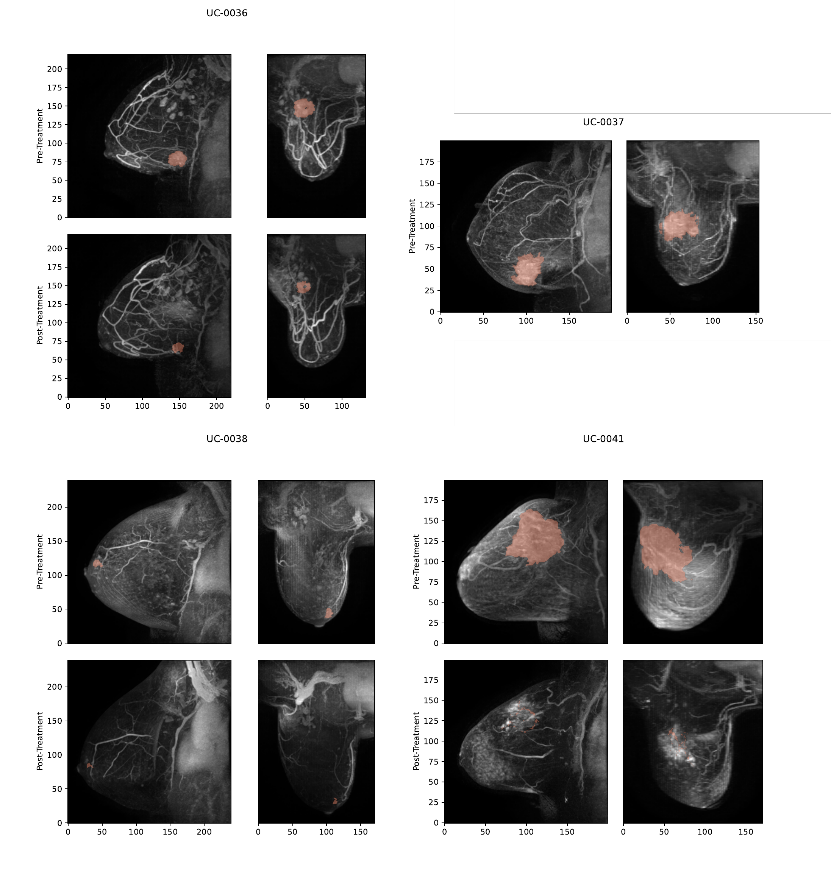


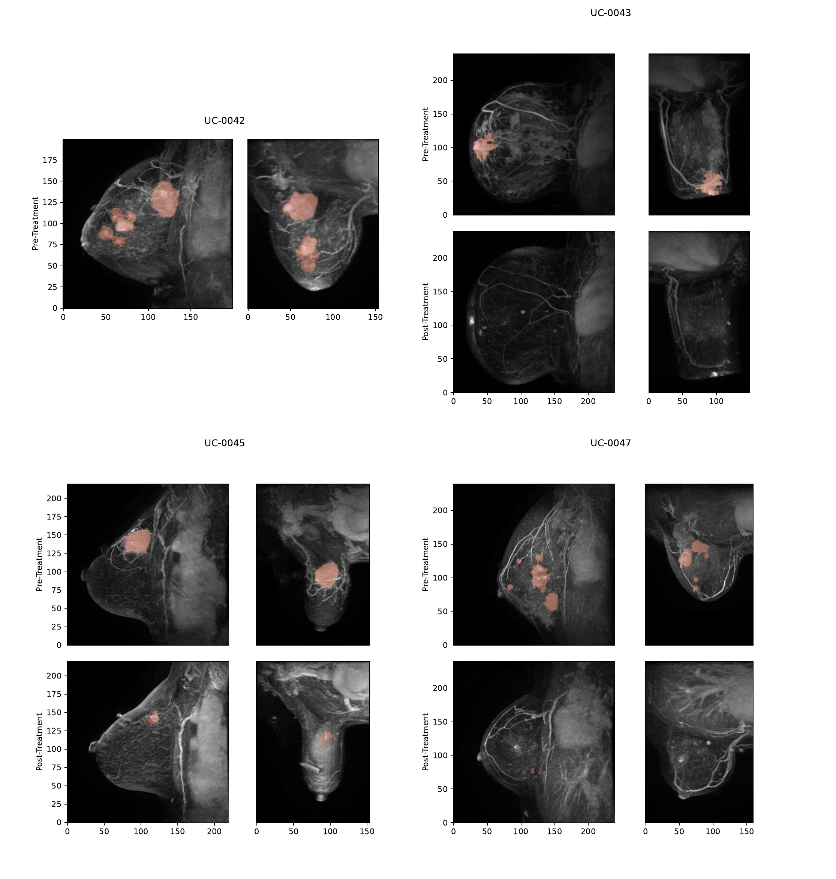


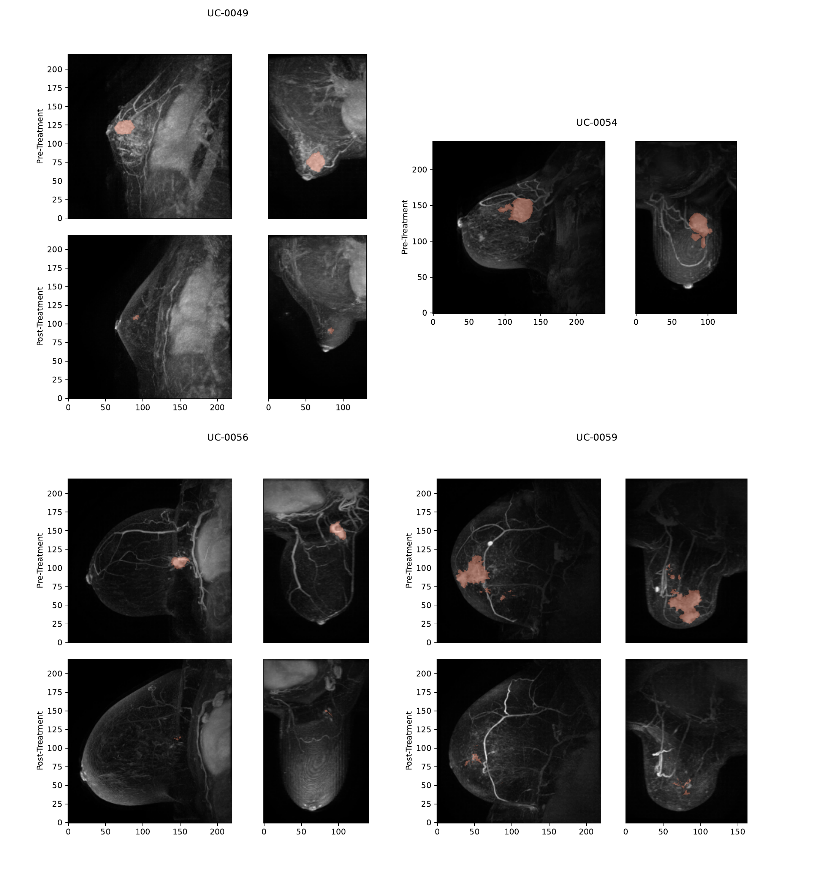


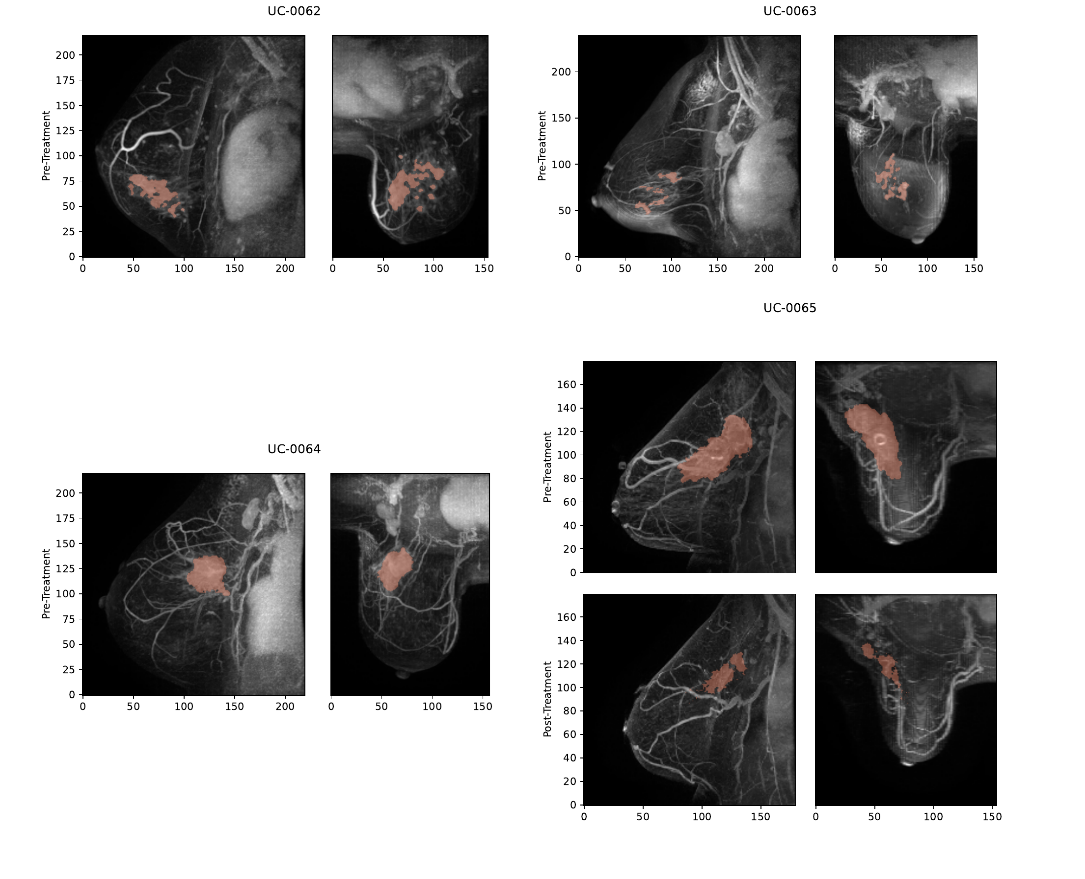


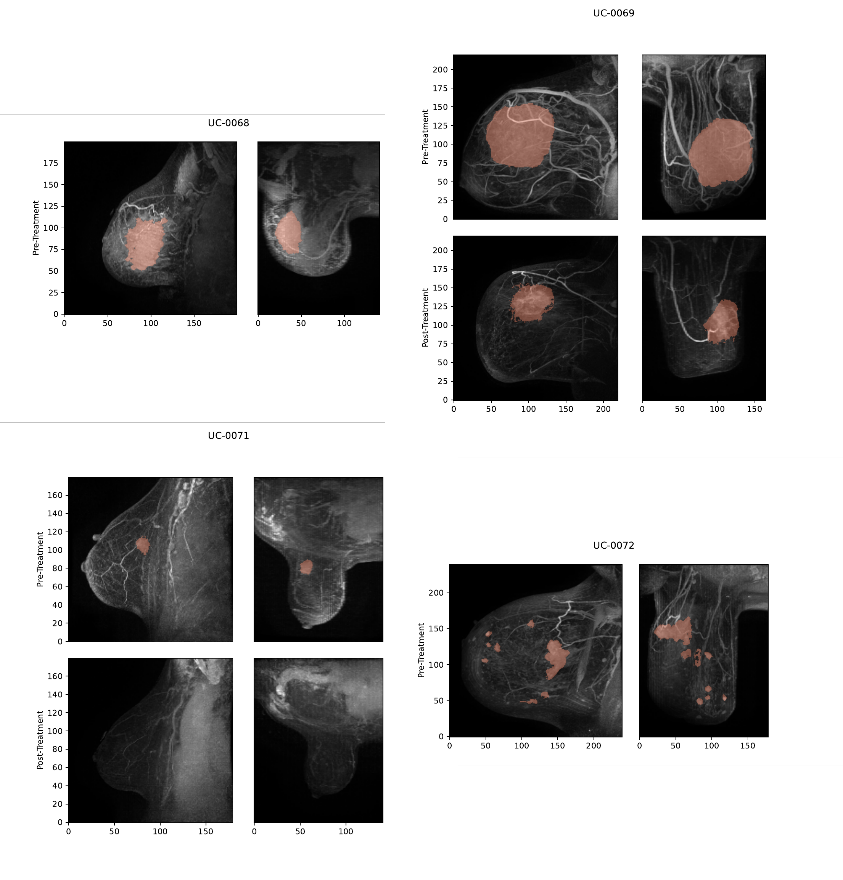


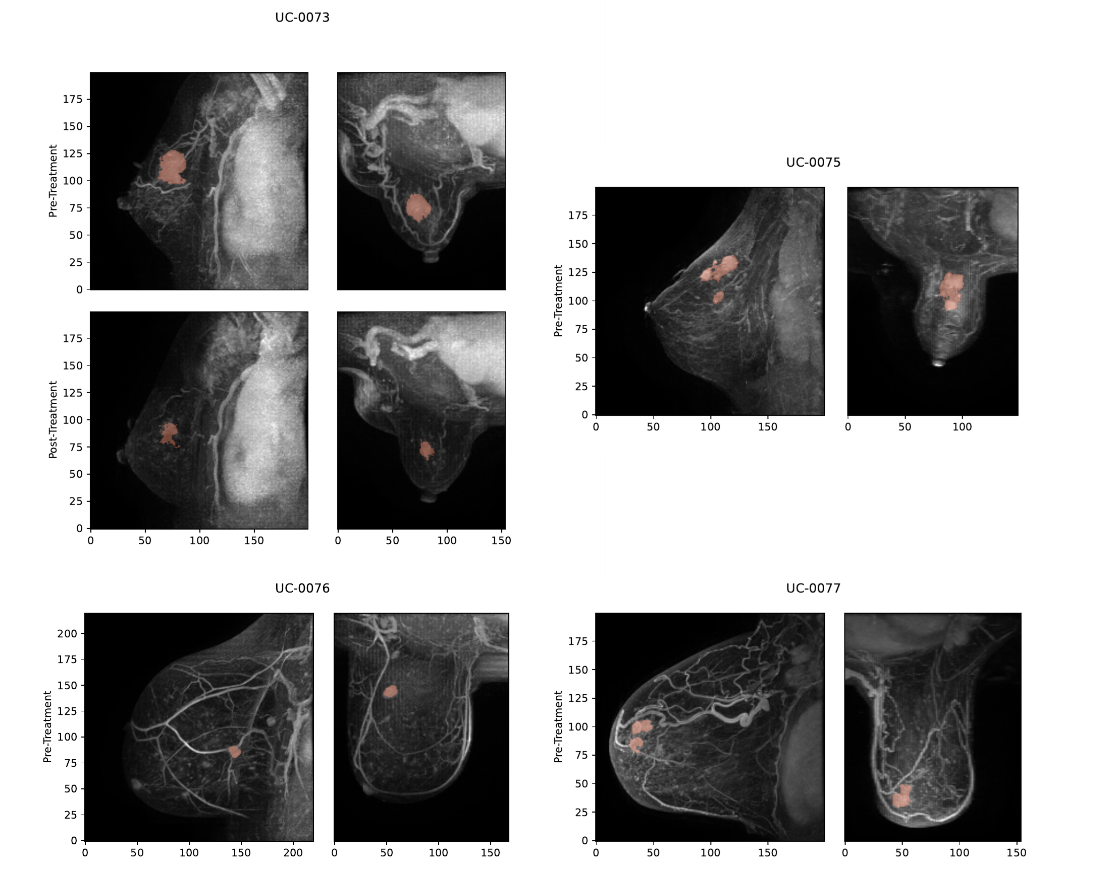


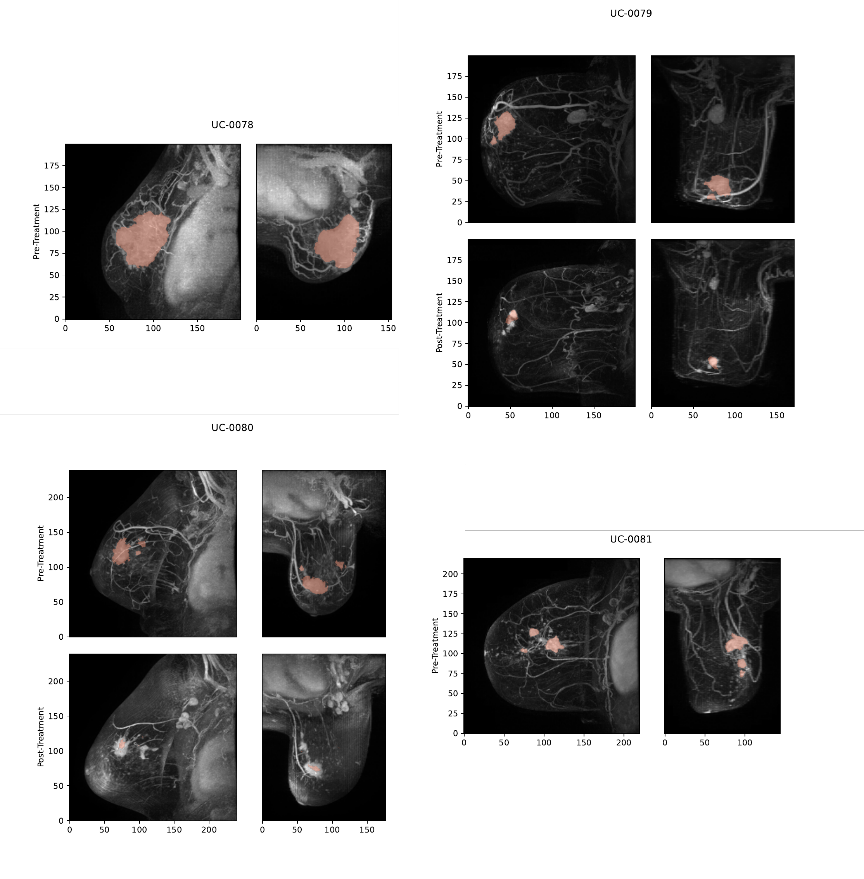

Supplement: Supplementary file 1 — Additional file 1. Supplementary Tables and Figures. [file 13058_2023_1654_MOESM1_ESM.docx]
